# ALE reveals a surprising link between [Fe-S] cluster formation, tryptophan biosynthesis and the potential regulatory protein TrpP in *Corynebacterium glutamicum*

**DOI:** 10.1186/s12866-025-03939-z

**Published:** 2025-04-14

**Authors:** Rico Zuchowski, Simone Schito, Christina Mack, Astrid Wirtz, Michael Bott, Tino Polen, Stephan Noack, Meike Baumgart

**Affiliations:** Institut für Bio- und Geowissenschaften, IBG-1: Biotechnologie, Forschungszentrum Jülich, Jülich, Germany

**Keywords:** Tryptophan, *Suf* operon, SufR, *Corynebacterium glutamicum*, Auxotrophy, [Fe-S] cluster, *Trp* operon, Adaptive laboratory evolution, ALE

## Abstract

**Background:**

The establishment of synthetic microbial communities comprising complementary auxotrophic strains requires efficient transport processes for common goods. With external supplementation of the required metabolite, most auxotrophic strains reach wild-type level growth. One exception was the l-trypton auxotrophic strain pha*Corynebacterium glutamicum* ΔTRP Δ*trpP*, which grew 35% slower than the wild type in supplemented defined media. *C. glutamicum* ΔTRP Δ*trpP* lacks the whole l-tryptophan biosynthesis cluster (TRP, cg3359-cg3364) as well as the putative l-tryptophan transporter TrpP (Cg3357). We wanted to explore the role of TrpP in l-tryptophan transport, metabolism or regulation and to elucidate the cause of growth limitation despite supplementation.

**Results:**

Mutants lacking either TRP or *trpP* revealed that the growth defect was caused solely by *trpP* deletion, whereas l-tryptophan auxotrophy was caused only by TRP deletion. Notably, not only the deletion but also the overexpression of *trpP* in an l-tryptophan producer increased the final l-tryptophan titer, arguing against a transport function of TrpP. A transcriptome comparison of *C. glutamicum* Δ*trpP* with the wild type showed alterations in the regulon of WhcA, that contains an [Fe-S] cluster. Through evolution-guided metabolic engineering, we discovered that inactivation of SufR (Cg1765) partially complemented the growth defect caused by Δ*trpP*. SufR is the transcriptional repressor of the *suf* operon (cg1764-cg1759), which encodes the only system of *C. glutamicum* for iron‒sulfur cluster formation and repair. Finally, we discovered that the combined deletion of *trpP* and *sufR* increased l-tryptophan production by almost 3-fold in comparison with the parental strain without the deletions.

**Conclusions:**

On the basis of our results, we exclude the possibility that TrpP is an l-tryptophan transporter. TrpP presence influences [Fe-S] cluster formation or repair, presumably through a regulatory function via direct interaction with another protein. [Fe-S] cluster availability influences not only certain enzymes but also targets of the WhiB-family regulator WhcA, which is involved in oxidative stress response. The reduced growth of WT Δ*trpP* is likely caused by the reduced activity of [Fe-S]-cluster-containing enzymes involved in central metabolism, such as aconitase or succinate: menaquinone oxidoreductase. In summary, we identified a very interesting link between l-tryptophan biosynthesis and iron sulfur cluster formation that is relevant for l-tryptophan production.

**Clinical trial number:**

Not applicable.

**Supplementary Information:**

The online version contains supplementary material available at 10.1186/s12866-025-03939-z.

## Introduction

*Corynebacterium glutamicum* is among the most important production organisms for industrial biotechnology, especially for amino acids and other small molecules, such as organic acids, diamines, vitamins and alcohols [1–4]. One important product is l-tryptophan, which is essential for humans and animals and is the least abundant amino acid in the cell with the most energy-demanding biosynthetic pathway [5]. In addition to its use as a food and feed additive [6], l-tryptophan is the precursor of other interesting products such as indole (fragrance) or halogenated substances for medical use [7–9]. Currently, fermentative or enzymatic methods are used for its production, with a total production volume of 41,000 metric tons in 2019, which is expected to increase further [10]. The production efficiency is, however, rather low in comparison with that of other amino acids, indicating the need to improve the microbial production efficiency [11].

In an approach to explore alternative production strategies, we designed and generated various synthetic co-cultures (CoNoS, Communities of Niche-optimized Strains) consisting of complementary *C. glutamicum* strains auxotrophic for certain amino acids [12, 13]. Most of the auxotrophic strains grew like the wild type in defined media supplemented with the required amino acid [13]. Interestingly, the l-tryptophan auxotrophic strain *C. glutamicum* WT* ΔTRP grew much slower than the wild type despite l-tryptophan supplementation, and the observed growth rate deviated significantly from the predicted growth rate [13]. *C. glutamicum* WT* ΔTRP (named from here onward WT ΔTRP Δ*trpP*) lacks the whole tryptophan biosynthesis operon *trpEGDCFBA* (*trp* operon) as well as the gene *trpP*, encoding a putative l-tryptophan transporter. Transposon mutagenesis studies revealed that l-tryptophan auxotrophy occurs if any of the *trp* genes is affected, except for *trpP* [14]. l-Tryptophan is taken up by *C. glutamicum* via AroP, but this is most likely not the only uptake system [15]. The annotation of TrpP as a putative l-tryptophan transporter was based on homology data to previously identified transporters and the fact that a DNA fragment containing *trpP* from an l-tryptophan-hyperproducing *C. glutamicum* strain rescued the 5-methyltryptophan (5-MT) sensitivity of *E. coli* [16, 17]. The selection of strains resistant to amino acid analogs is a traditional method to isolate amino acid overproducers. Heery and Dunican already reported that the observed phenotype does not fit to the model of TrpP being an l-tryptophan import system and that TrpP might have a regulatory function via the titration of a DNA binding factor [16]. Furthermore, the sequence identity of TrpP to the mentioned transporters is rather low (17–22%) and with current blast search tools (NCBI BLAST) or searches in protein family databases (Interpro) we did not get any similar proteins that have transporter function. TrpP has only three transmembrane helices, whereas transporters usually have twelve transmembrane helices. Thus, from today’s perspective, a transporter function of TrpP seems rather unlikely. A recent study suggested that TrpP (there named Cgl1) is similar to the SdpI protein of *Bacillus subtilis* [18]. *B. subtilis* SdpI belongs to a three-protein signal-transduction pathway consisting of SdpC, SdpI and SdpR, which governs immunity to a protein toxin involved in cannibalism during endospore formation [19, 20].

In this study, we wanted to explore the potential role of TrpP in l-tryptophan transport, metabolism or regulation and to elucidate the cause of the growth limitation of *C. glutamicum* WT ΔTRP Δ*trpP*. We identified *trpP* as a nonessential but highly relevant gene for cell growth, with a surprising link to the [Fe-S]-cluster assembly machinery found via an evolution-guided approach. Interestingly, the deletion of *trpP*, both alone and in combination with *sufR*, significantly improved l-tryptophan production. Thus, we also discovered a new l-tryptophan production trait for *C. glutamicum*.

## Materials and methods

### Bacterial strains, plasmids and growth conditions

All strains used in this study are listed in Table 1 and are based on the wild-type *C. glutamicum* ATCC13032 or on *C. glutamicum* C1*, a genome-reduced variant [21]. Strain cultivation was performed as described previously [13]. *Escherichia coli* cultivation was performed in liquid lysogeny broth (LB) media or on agar plates [22] at 37 °C. For *E. coli* strains harboring plasmids, 50 µg mL^− 1^ kanamycin was added to the medium. For cultivating *C. glutamicum* strains, complex brain heart infusion (BHI) medium (Difco Laboratories, Detroit, USA) or defined CGXII medium [23] with a protocatechuic acid concentration of 0.03 g L^− 1^ was used at 30 °C. Kanamycin (25 µg mL^− 1^ final concentration) was added to the medium for plasmid carrying *C. glutamicum* strains, and different amounts of isopropyl β-d-1-thiogalactopyranoside (IPTG) were added for the pPREx2-plasmid system to induce expression [24].

**Table 1:** Bacterial strains and plasmids used in this study

Strain or plasmid	Relevant characteristics	Source or Reference
**E. coli**		
DH5α	F^−^ Φ80*dlac*∆(*lacZ*)M15 ∆(*lacZYA-argF*) U169 *endA1 recA1 hsdR17* (r_K_^−^, m_K_^+^) *deoR thi-1 phoA supE44* λ^−^*gyrA96 relA1*; strain used for cloning procedures	[26]
***C. glutamicum***		
ATCC13032 (WT)	Biotin-auxotrophic wild type	[78]
WT ΔTRP Δ*trpP* (WT* ΔTRP in the original publication)	WT with an in-frame deletion of Δ*trpP* (cg3357) Δ*trpE* (cg3359) Δ*trpG* (cg3360) Δ*trpD* (cg3361) Δ*trpCF* (cg3362) Δ*trpB* (cg3363) Δ*trpA* (cg3364)	[13]
C1* ΔTRP Δ*trpP* (C1* ΔTRP in the original publication)	C1* with an in-frame deletion of Δ*trpP* (cg3357) Δ*trpE* (cg3359) Δ*trpG* (cg3360) Δ*trpD* (cg3361) Δ*trpCF* (cg3362) Δ*trpB* (cg3363) Δ*trpA* (cg3364)	[13]
C1* ΔTRP Δ*trpP* evo1	Evolved C1* ΔTRP Δ*trpP* strain number 1 with mutation SufR_L25P_	This study
C1* ΔTRP Δ*trpP* evo2	Evolved C1* ΔTRP Δ*trpP* strain number 2 with mutation SufR_R77G_	This study
C1* ΔTRP Δ*trpP* evo3	Evolved C1* ΔTRP Δ*trpP* strain number 3 with mutation SufR_Q193*_	This study
WT Δ*trpP*	WT with an in-frame deletion of Δ*trpP* (cg3357)	This study
WT ΔTRP	WT with an in-frame deletion of Δ*trpE* (cg3359) Δ*trpG* (cg3360) Δ*trpD* (cg3361) Δ*trpCF* (cg3362) Δ*trpB* (cg3363) Δ*trpA* (cg3364)	This study
WT ΔTRP Δ*trpP* Δ*sufR*	WT ΔTRP Δ*trpP* with a partial deletion of *sufR* (cg1765) until the *sufB* (cg1764) promoter site with an inserted stop codon at the end of *sufR*	This study
WT ΔTRP Δ*trpP* SufR_L25P_	WT ΔTRP Δ*trpP* with point mutation L25P in SufR	This study
WT ΔTRP Δ*trpP* SufR_Q193*_	WT ΔTRP Δ*trpP* with point mutation Q193* in SufR	This study
WT ΔTRP Δ*trpP* ΔP_*sufB*_::P_*tuf*_	WT ΔTRP Δ*trpP* with an exchange of *sufR* (cg1765) and the native *sufB* promoter sequence with the *tuf* promoter (cg0587)	This study
WT ΔTRP Δ*trpP* ΔP_*sufB*_::P_*dapA*_	WT ΔTRP Δ*trpP* with an in frame exchange of *sufR* (cg1765) including the native *sufB* promoter sequence with the *dapA* promoter (cg2161)	This study
WT Δ*trpP* Δ*sufR*	WT Δ*trpP* with an partial deletion of *sufR* (cg1765) until the *sufB* (cg1764) promoter site with an inserted stop codon at the end of *sufR*	This study
WT TRP^+^	WT with point mutations *trpL*_fbr_ (with mutation of the third of three tandem Trp codons of *trpL* TGG ➜ TGA) and TrpE_S38R_ (Cg3359)	This study
WT TRP^++^	WT TRP^+^with point mutation TrpD_A162E_ (Cg3361)	This study
WT TRP^+++^	WT TRP^++^ with in-frame deletion of *aroP* (cg1257)	This study
WT TRP^+++^ Δ*sufR*	WT TRP^+++^ with a partial deletion of *sufR* (cg1765) until the *sufB* (cg1764) promoter site with an inserted stop codon at the end of *sufR*	This study
WT TRP^+++^ Δ*trpP*	WT TRP^+++^ with an in-frame deletion of *trpP* (cg3357)	This study
WT TRP^+++^ Δ*trpP* Δ*sufR*	WT TRP^+++^ Δ*trpP* with an partial deletion of *sufR* (cg1765) until the *sufB* (cg1764) promoter site with an inserted stop codon at the end of *sufR*	This study
**Plasmids**		
pK19*mobsacB*	Kan^R^.; plasmid for allelic exchange in *C. glutamicum*; (pK18 *ori*V_*E.c*_., *sacB*, *lacZ*α)	[27]
pK19*mobsacB*-TrpL_fbr_ TrpE_S38R_	Kan^R^.; pK19*mobsacB* derivative for mutation of *trpL* (TGG◊TGA) and TrpE (Cg3359) S38R in *C. glutamicum*	[13]
pK19*mobsacB*-ΔTRPv2_(*trpP)*	Kan^R^.; pK19*mobsacB* derivative for in-frame deletion of TRP-operon (cg3359-cg3364) without disrupting *trpP* (cg3357) in *C. glutamicum*	This study
pK19*mobsacB*-Δ*trpPv2* (TrpL_fbr_ TrpE_S38R_)	Kan^R^.; pK19*mobsacB* derivative for in-frame deletion of *trpP* (cg3357) in *C. glutamicum* WT TRP^+^without disrupting the inserted mutations in *trpL* and TrpE (Cg3359)	This study
pK19*mobsacB*-Δ*trpP*	Kan^R^.; pK19*mobsacB* derivative for in-frame deletion of *trpP* (cg3357) in *C. glutamicum*	This study
pK19*mobsacB*-Δ*aroP*	Kan^R^.; pK19*mobsacB* derivative for in-frame deletion of *aroP* (cg1257) in *C. glutamicum*	This study
pK19*mobsacB*-Δ*sufR*	Kan^R^.; pK19*mobsacB* derivative for partial in-frame deletion of *sufR* (cg1765) with an inserted stop codon at the end of *sufR* in *C. glutamicum*	This study
pK19*mobsacB*-SufR_L25P_	Kan^R^.; pK19*mobsacB* derivative for mutation of SufR (Cg1765) L25P in *C. glutamicum*	This study
pK19*mobsacB*-SufR_Q193*_	Kan^R^.; pK19*mobsacB* derivative mutation of SufR (Cg1765) Q193* in *C. glutamicum*	This study
pK19*mobsacB*-TrpD_A162E_	Kan^R^.; pK19*mobsacB* derivative for mutation of TrpD (Cg3361) A162E in *C. glutamicum*	This study
pK19*mobsacB*-Δ*sufR*ΔP_*sufB*_::P_*tuf*_	Kan^R^.; pK19*mobsacB* derivative for deletion of *sufR* (cg1765) with integration of P_*tuf*_ (cg0587) in front of the SUF-cluster in *C. glutamicum*	This study
pK19*mobsacB*-Δ*sufR*ΔP_*sufB*_::P_*dapA*_	Kan^R^.; pK19*mobsacB* derivative for deletion of *sufR* (cg1765) with integration of P_*tuf*_ (cg2161) in front of the SUF-cluster in *C. glutamicum*	This study
pPREx2	Kan^R^; *C. glutamicum*/*E. coli* shuttle vector for regulated gene expression using the P_*tac*_ promoter	[24]
pPREx2-*trpP*	Kan^R^; pPREx2 derivative for *trpP* (cg3357) expression under control of P_*tac*_	This study
pJC1	Kan^R^; *E. coli*/*C. glutamicum* shuttle (oriV_*E*.*coli*_, oriV_*C.glutamicum*_)	[28]
pJC1-Venus-Term	Kan^R^; pJC1 derivative carrying the venus coding sequence and additional terminators, used as PCR template for venus.	[79]
pJC1-P_*sufR*_-venus	Kan^R^; pJC1 derivative with venus fluorescent protein under control of the native *sufR* (cg1765) promoter	This study
pJC1-P_*sufR*_-venus-Mut_TSS1_	Kan^R^; pJC1 derivative with venus fluorescent protein under control of the *sufR* (cg1765) promoter with mutated − 10 region of TSS1 (AAC ◊ CTG)	This study
pJC1-P_*sufR*_-venus-Mut_TSS2_	Kan^R^; pJC1 derivative with venus fluorescent protein under control of the *sufR* (cg1765) promoter with mutated − 10 region of TSS2 (GTG ◊ TAC)	This study

### Microscale and 50 ml scale cultivation

Unless otherwise stated, detailed characterization of single-strain growth behavior was performed via microscale (800 µL) cultivation in a BioLector (Beckman Coulter, Krefeld, Germany) as described previously [13]. A first preculture was prepared in BHI medium by inoculation with a single colony from a BHI plate and cultivated at 30 °C and 900 rpm for 8 h. Sedimented cells (4000*g*, 10 min) from this culture were used to inoculate a second preculture in CGXII medium with 111 mM glucose and 0.5 mM l-tryptophan if necessary and cultivated overnight at 30 °C and 900 rpm. The main cultures were inoculated from cells pelleted and suspended in sterile PBS (phosphate-buffered saline, 137 mM NaCl, 2.7 mM KCl, 10 mM Na_2_HPO_4_, 1.8 mM KH_2_PO_4_, pH 7.4 with HCl) and cultivated in 48-well Flowerplates (Beckman Coulter, Krefeld, Germany) at 30 °C, 1400 rpm, and 85% humidity. The Python package Bletl [25] was employed for growth rate determination as described previously [13]. For strains harboring the venus-fusion constructs, fluorescence was additionally measured with an eYFP filter (Ex 508 nm, Em 532 nm).

To analyze amino acid production, main cultures were prepared from second precultures as described above by inoculation of 50 ml CGXII medium with 111 mM glucose to a starting OD_600_ of 0.8 in 500 ml baffled shake flasks. The main cultures were incubated at 30 °C, 130 rpm, and 85% humidity in a Minitron shaker (Infors HT, Einsbach, Germany). Samples were taken manually at defined time points, and the cell-free supernatants were analyzed by HPLC.

### Recombinant DNA work (and construction of deletion mutants)

All plasmids used in this study are listed in Table 1. All oligonucleotides used in this study are listed in Table S1. *Escherichia coli* DH5α was used as host for cloning [26]. To construct strains with genomic deletions or mutations, the pK19*mobacB* system was used [27]. For plasmid-based gene expression, the pPREx2 system was used [24]. For the promoter fusion studies, the pJC1 system [28] was used.

### Adaptive laboratory evolution (ALE) and genome sequencing

For strain evolution, the cells were cultivated in repetitive-batch mode using the Mini Pilot Plant as described previously [29, 30]. In a 48-well Flowerplate, three wells with CGXII medium (111 mM glucose, 0.5 g L^− 1^l-tryptophan) were initially inoculated with C1*ΔTRP Δ*trpP* to an OD_600_ of 0.5 and cultivated until a defined backscatter (BS) threshold of BS = 25 was reached that triggered the automated transfer of 50 µl of the grown culture into the next well filled with 800 µl CGXII medium. 16 repetitive batches were performed in this fashion and the resulting raw backscatter data were processed for estimation of specific growth rates as described previously [13]. From the last batch, cell material was streaked on a BHI plate. Single clones from this plate were retested upon growth in the BioLector. For each initial replicate in the ALE experiment, the gDNA of one well with a strain showing an improved growth rate in comparison to the C1*ΔTRP Δ*trpP* strain was isolated. DNA isolation and whole-genome sequencing were performed as described previously [31]. The gDNA was isolated with the DNeasy Blood & Tissue Kit (Qiagen, Hilden, Germany) and used for library preparation with the NEBNext^®^ Ultra™ II DNA Library Prep Kit (NEB, Frankfurt am Main, Germany). After the library was evaluated via qPCR (KAPA library quantification kit, Peqlab, Erlangen, Germany), it was normalized by pooling and subjected to in-house paired-end sequencing (read length of 2 × 150 bases, MiSeq, Illumina^®^). The resulting data were processed with CLC Genomic Workbench software (Qiagen, Hilden, Germany) and mapped to the genome sequence of *C. glutamicum* C1 (CP017995). The data for this study have been deposited in the European Nucleotide Archive (ENA) at EMBL-EBI under accession number PRJEB76054 (https://www.ebi.ac.uk/ena/browser/view/PRJEB76054).

### Supernatant analysis

Analysis of the concentrations of l-tryptophan and other amino acids in the supernatant was performed with the same method as described before [31] via an uHPLC system (Agilent 1290 Infinity, Agilent Technologies, Santa Clara, CA). A reversed-phase column (Kinetex 2.6 μm EVO C18 100 Å, 100 × 2.1 mm) was used as the stationary phase together with a precolumn (Phenomenex, SecurityGuard™ ULTRA C18, sub 2 μm, 2.1 mm internal diameter). For the mobile phase, a gradient of buffer A (10 mM Na_2_HPO_4_ (anhydr.), 10 mM Na_2_B_4_O_7_ × 10 H_2_O, pH 8.2 with HCl) and buffer B (methanol) with a flow rate of 0.42 mL min^− 1^ was used. Amino acid quantification was performed relative to amino acid standards which were measured before and after each run.

### Global gene expression analysis via DNA microarrays

For analysis of the gene expression profile of *C. glutamicum*, cells were first cultivated with two precultures and a 50 mL main culture in CGXII as described above to an OD_600_ of 5. The cells were harvested and used for RNA isolation with the RNeasy MiniKit (QIAGEN, Hilden, Germany) as described elsewhere [31]. The concentration of the resulting RNA was determined at 260 nm with a Colibri Microvolume Spectrometer (Titertek-Berthold, Germany). Afterward, cDNA was synthesized from 15 µg RNA with random hexamer primers and SuperScript III reverse transcriptase (Life Technologies, Darmstadt, Germany) as described previously [32, 33]. For fluorescent labeling, the nucleotide analogs Cy3-dUTP or Cy5-dUTP (GE Healthcare, Eindhoven, Netherlands) were used. The probes were purified via Amicon Centrifugal Filters (Merck Millipore, Darmstadt, Germany), pooled and hybridized for 17 h at 65 °C on custom-made 4 × 44 K 60mer DNA microarrays (Agilent Technologies, Waldbronn, Germany) using Agilent’s Gene Expression Hybridization Kit and Hybridization Chamber. To avoid batch effects due to similar labeling, a color swap was performed for two out of four arrays. After the arrays were washed with the Agilent wash buffer kit, the fluorescence of the arrays was measured at 532 nm (Cy3-dUTP) and 635 nm (Cy5-dUTP) at 5 mm resolution with a GenePix 4000B laser scanner operated by GenePix Pro 7.0 software (Molecular Devices, Sunnyvale, USA) and analyzed as described previously [32] with BioConductor R-packages limma and marray (http://www.bioconductor.org). The full microarray datasets of this study have been deposited in the NCBI Gene Expression Omnibus and can be found under the GEO accession number GSE272128.

## Results

### Influence of TrpP and the TRP operon on growth

**Fig. 1:**
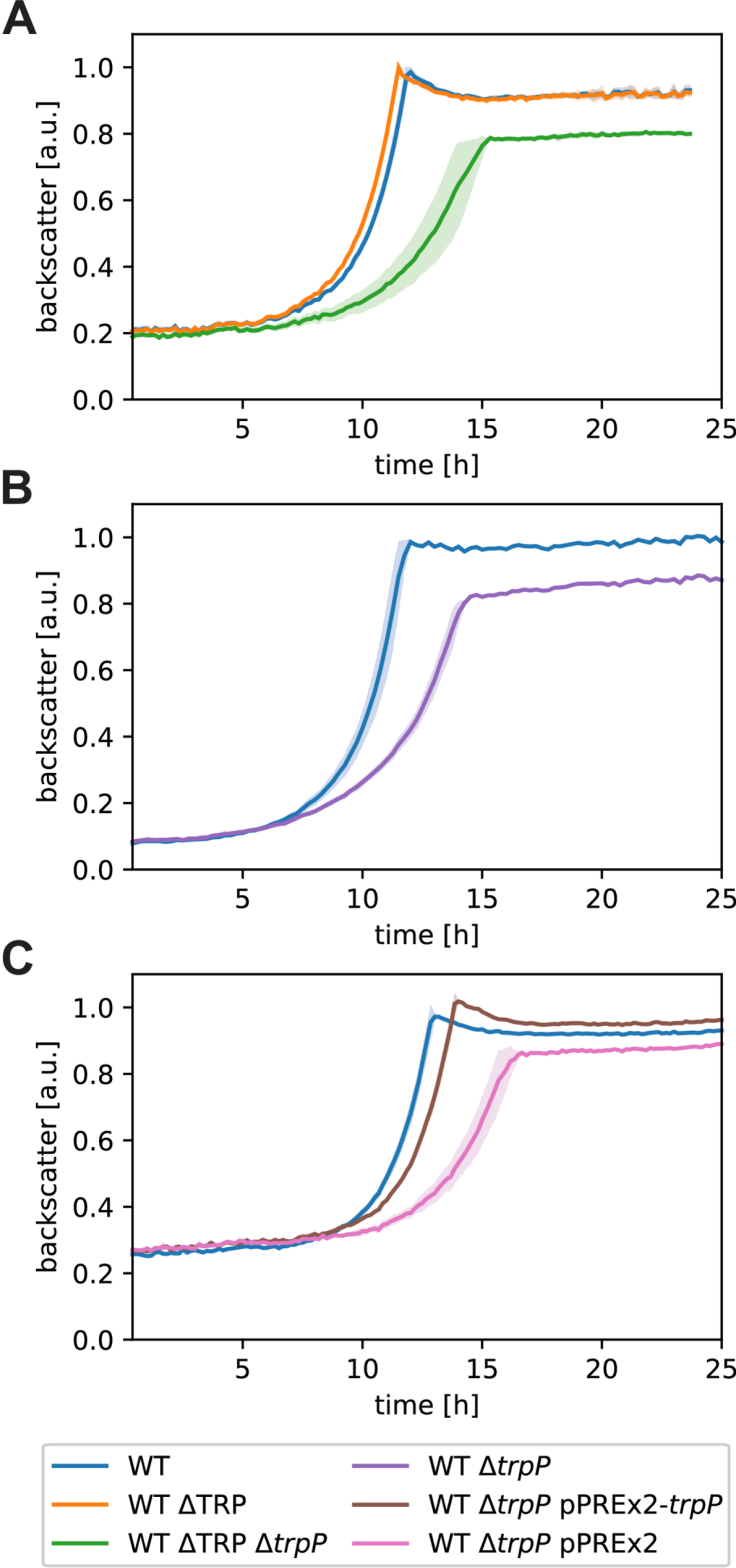
Growth of the *trp* operon and *trpP* deletion and complemented strains. (**A**) Growth of the WT and two l-tryptophan auxotrophic strains. (**B**) Growth of WT and WT Δ*trpP*. (**C**) Plasmid-based complementation of WT Δ*trpP* with pPREx2-*trpP.* All cultures were grown in defined CGXII media supplemented with 111 mM d-glucose and 0.5 mM l-tryptophan when necessary. Kanamycin (25 µg mL^− 1^) was added for strains harboring a plasmid. The backscatter data were normalized based on the maximum value recorded for each WT in the corresponding experiment. The mean values of biological triplicates are shown as lines and standard deviations are shown as shaded areas

**Fig. 2:**
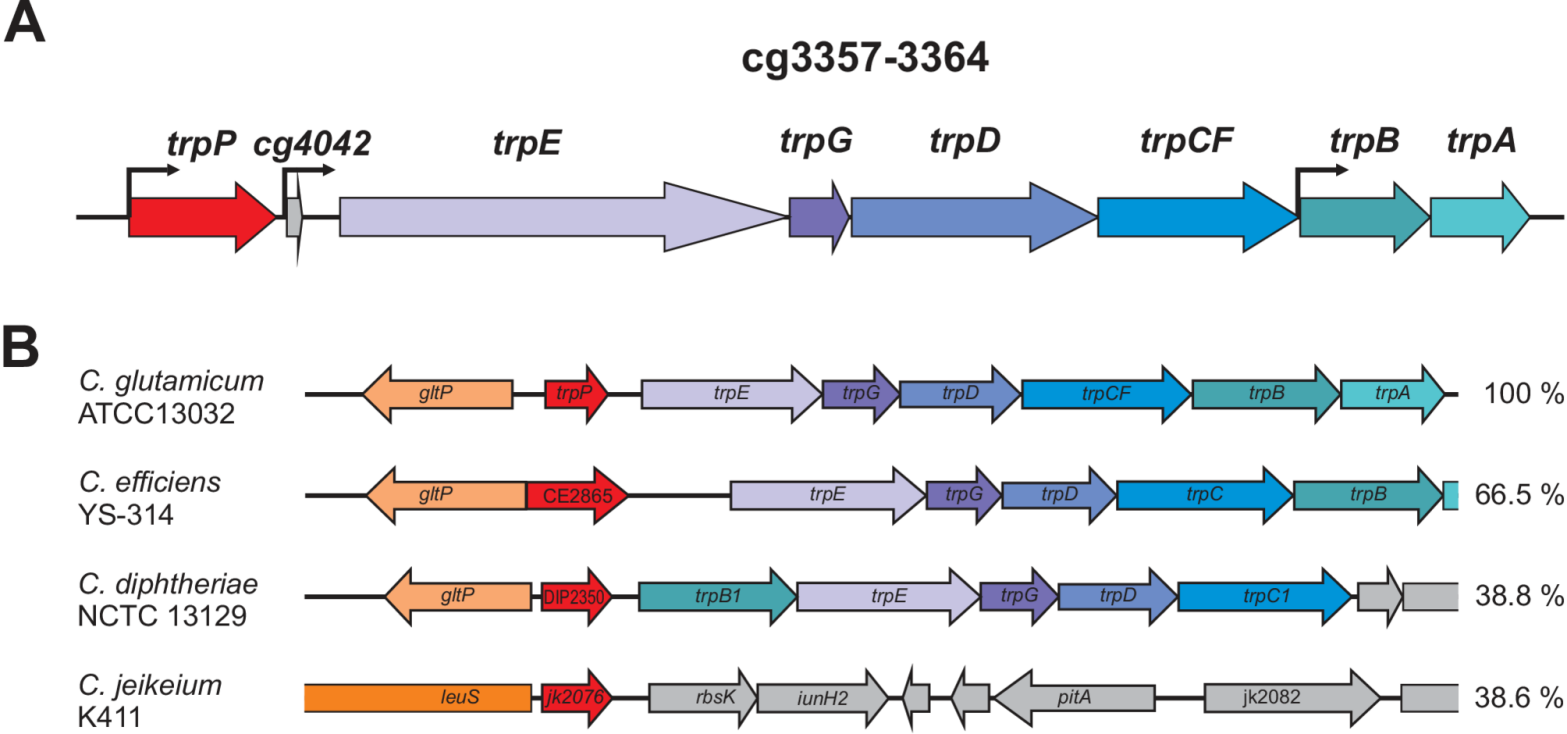
Transcriptional organization and genomic context of *trpP* and the *trp* operon. (**A**) Transcriptional organization of *trpP* and the l-tryptophan synthesis operon (*trp* operon). The arrows represent transcriptional start sites according to [34]. (**B**) Genomic organization of *trpP*-TRP in *C. glutamcium* and related organisms. The numbers on the right represent the percent amino acid sequence identity of the TrpP homologs to *C. glutamicum* TrpP according to Clustal Omega [37]. Data were taken from MicrobesOnline [38], and genes were drawn approximately to scale

In this study, we wanted to investigate the potential role of TrpP in l-tryptophan transport, synthesis, or regulation and to elucidate the cause of the growth limitation of an l-tryptophan auxotrophic strain. During our CoNoS project, we observed that even with sufficient external l-tryptophan supplementation, both the growth rate and the final backscatter of *C. glutamicum* WT* ΔTRP (named here WT ΔTRP Δ*trpP*) were significantly lower than those of the wild type [13] (Fig. 1A). In WT ΔTRP Δ*trpP*, the whole l-tryptophan biosynthesis operon and *trpP* (cg3357), encoding a putative l-tryptophan transporter, were deleted [13] (Fig. 2A). In this study, we refer to the operon *trpEGDCFBA* (cg3359-cg3364) as the *trp* operon and to cg3357 as *trpP*.

First, we wanted to clarify whether the growth defect was caused by deletion of the *trp* operon, deletion of *trpP* or by an additive effect of the double deletion. We generated two strains, WT ΔTRP (lacking the *trp* operon but still containing *trpP*, l-tryptophan auxotrophic) and WT Δ*trpP* (lacking only *trpP*, not l-tryptophan auxotrophic), and analyzed their growth behavior. The strain WT ΔTRP grew very similarly to the wild type (Fig. 1A), whereas WT Δ*trpP* reached only 67% of the growth rate and 83% of the final backscatter in comparison to the WT (Fig. 1B). These results indicated that the observed growth defect of WT ΔTRP Δ*trpP* was caused solely by the deletion of *trpP*.

To exclude that the observed growth defect of WT Δ*trpP* was caused by any unwanted mutation, we tested whether the growth defect can be complemented with plasmid-based expression of *trpP*. The strain WT Δ*trpP* pPREx2-*trpP* grew very similarly to the WT (95% of WT growth rate) without the addition of IPTG to induce *trpP* expression (leaky *trpP* expression) (Fig. 1C). The addition of IPTG did not increase growth further. Thus, we assume that the growth defect of WT ΔTRP Δ*trpP* was indeed caused by the deletion of *trpP*.

To test whether the growth defect of WT Δ*trpP* is dependent on the medium, we tested growth in complex media and in defined media with different carbon sources. Under all tested conditions, a clear growth defect of WT Δ*trpP* in comparison to WT was observed with both the growth rate and final backscatter being reduced (Fig. S1). The growth defect of WT Δ*trpP* was the smallest in BHI complex medium, whereas in CGXII with carbon sources such as gluconate or *myo*-inositol, the lag phase of WT Δ*trpP* was prolonged. Notably, when acetate was used as carbon source, an initial acetate concentration of 400 mM completely inhibited the growth of WT Δ*trpP*, whereas the WT grew after a prolonged lag phase. In summary, the growth defect of WT Δ*trpP* was present under all tested conditions and was even worse with acetate.

### In silico analysis of TrpP and its surrounding genome region

We performed a detailed in silico analysis of the *trpP* gene and the resulting protein TrpP to identify possible functions. First, we analyzed the genomic context of *trpP* and its conservation because genes with a functional link often cluster together across several organisms. In the *C. glutamicum* genome, *trpP* is located next to the *trp* operon, encoding the genes for l-tryptophan biosynthesis (Fig. 2A). *trpP* has a transcriptional start site (TSS) that is identical with the TrpP start codon [34]; thus, it is one of the many leaderless transcripts of *C. glutamicum*. The *trp* operon is presumably transcribed from a separate TSS in front of the leader peptide cg4042 (*trpL*) [34, 35]. A further internal TSS was identified at the start codon of cg3363 (*trpB*) [34] (Fig. 2A). The genes of the *trp* operon usually cluster together across many organisms. The genomic localization of *trpP* next to the *trp* operon is similar in closely related organisms, such as *Corynebacterium efficiens*, *Corynebacterium diphtheriae* (Fig. 2B) and *Corynebacterium ulcerans* [36]. In more distantly related Corynebacteria, such as *Corynebacterium jeikeium*, *trpP* is often located in the neighborhood of the leucyl-tRNA-synthetase gene *leuS*. There are only a few examples of proteins homologous to TrpP in mycobacteria, one example is from *Mycobacterium avium* (accession: PBJ41361).

**Fig. 3:**
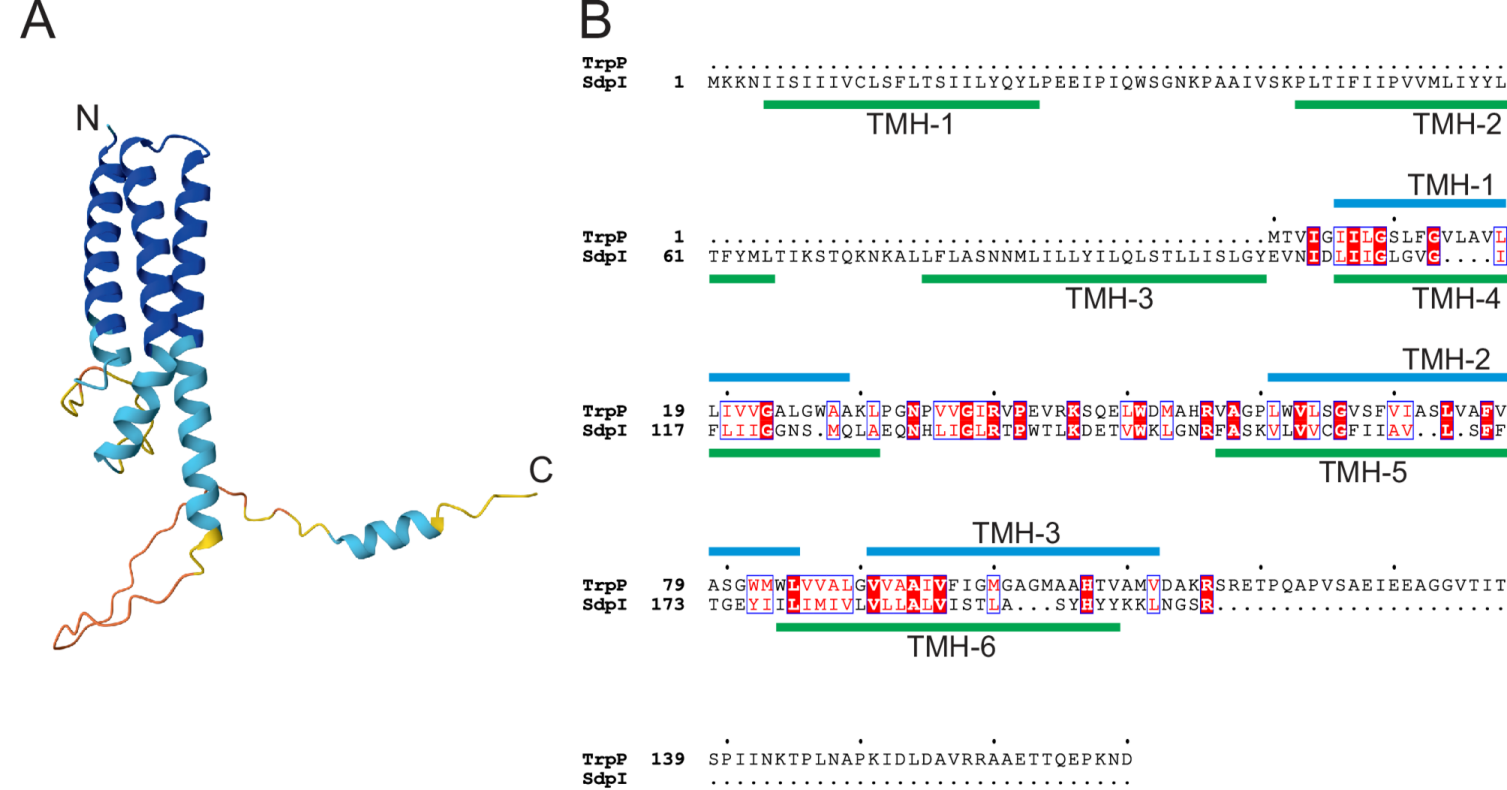
AlphaFold model of TrpP and alignment with SdpI. (**A**) AlphaFold model of TrpP. N and C indicate the N- and C-termini of the protein, respectively. The N-terminus is predicted to be extracellular, and the C-terminus is predicted to be intracellular. The protein chain color represents the model confidence from dark blue (very high), light blue (high) and yellow (low) to red (very low) [40, 41]. (**B**) Sequence alignment of *C. glutamicum* TrpP and *B. subtilis* SdpI, prepared with Clustal Omega [42] and visualized with ESPribt 3.0 [43]. Transmembrane helices according to Phobius [44] are given for TrpP in blue and for SdpI in green. Residues with high similarity are marked with red letters, and identical residues are marked with a red background

TrpP is a protein of 170 amino acids with a theoretical molecular mass of approximately 17.7 kDa and a pI of 6.73. According to several topology prediction tools, it has three transmembrane (TM) helices with the N-terminus in the periplasm and the C-terminus in the cytoplasm (Fig. 3, Fig. S2, Table S2). According to Interpro, TrpP belongs to the SdpI/YhfL protein family whose members are annotated as membrane proteins of unknown function, l-tryptophan permease or SdpI/YhfL family proteins [39]. The description of the SdpI/YhfL protein family (IPR025962) is based on one publication about a three-protein signaling pathway in *Bacillus subtilis* [19] and a theoretical work elucidating the sequence space of SdpI family proteins (TrpP is named Cgl1 in this study) [18]. SdpI has six TM helices, where helices 1–3 bind the exported toxic protein SdpC and helices 4–6 bind the cytoplasmic transcriptional repressor SdpR [19]. There are no homologs of SdpC or SdpR in *C. glutamicum*. TrpP aligns with SdpI in the C-terminal region with an amino acid identity of 27%. These findings suggest that TrpP interacts with another protein and thereby performs a regulatory function. While SdpI family proteins are generally present in many organisms, proteins reasonably similar to TrpP are mostly found in *Corynebacteria*, *Rhodococcus* or *Nocardia* (Fig. 2B, Fig. S3). *C. glutamicum* contains a second SdpI protein in addition to TrpP, which is encoded by cg0900. This protein also contains three TM helices that align with TM1, 5 and 6 of SdpI (Fig. S4). SdpI and Cg0900 contain 15.6% of identical amino acids. TrpP and Cg0900 contain 24.8% of identical amino acids. Further information on the SdpI protein family is available at [18].

According to the current knowledge, there are three theories regarding TrpP function: (i) TrpP has a transporter function, but this is based on an old annotation and rather unlikely due to just three transmembrane helices, (ii) TrpP is a membrane protein with potential regulatory function by interaction with another protein. In this case, the C-terminal extension might influence TrpP activity, e.g. by binding to a possible ligand. (iii) Entirely different functions to i) and ii). In the following, we describe our strategy to obtain a better understanding of TrpP function.

### Influence of *trpP* deletion and overexpression on l-tryptophan production

As described in the introduction, TrpP was annotated as a potential l-tryptophan transporter owing to its homology to other transporters and its neighborhood with l-tryptophan synthesis genes in the genome [16]. However, this function remains questionable. In general, transporters can have a strong influence on production outcomes because they are either responsible for efficient export of the product out of the cell or reduce the product titer due to reuptake of the product. Since it was not clear whether transport is the real function of TrpP and if it is either an importer or an exporter, we performed an experiment with the l-tryptophan producing strain WT TRP^+++^. This strain harbors the following mutations: TrpE_S38R_ and TrpD_A162E_, which make these two enzymes feedback resistant; mutation of the third of three tandem Trp codons in the leader peptide TrpL to increase translation in the presence of l-tryptophan; and deletion of the main l-tryptophan importer AroP to avoid reuptake of the product. If TrpP is an l-tryptophan exporter, the deletion should reduce l-tryptophan production, and the overexpression should increase l-tryptophan production. If TrpP is an l-tryptophan importer, its deletion should increase l-tryptophan production and its overexpression should decrease l-tryptophan production.

**Fig. 4:**
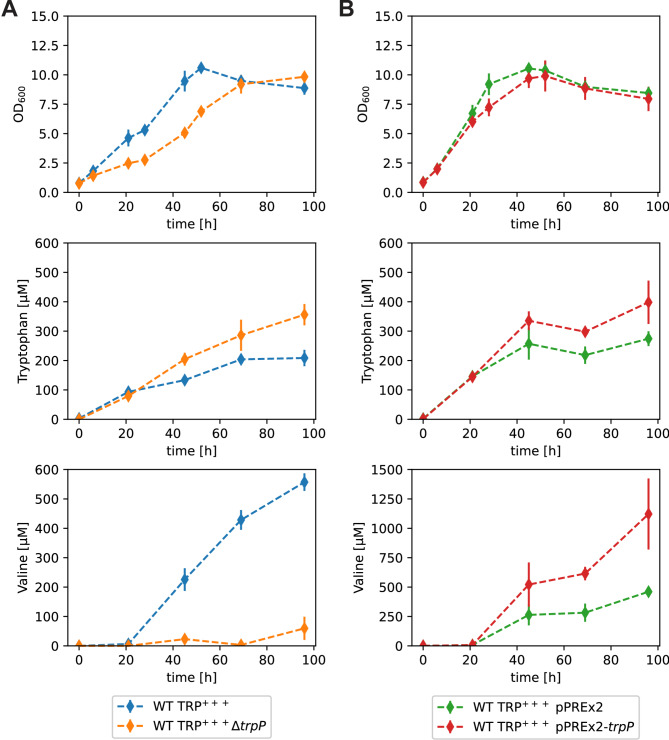
Characterization of the influence of *trpP* on amino acid formation in *C. glutamicum*. Cultivation of WT TRP^+++^-derived *trpP* deletion (**A**) and plasmid-based overexpression (**B**) strains regarding their growth, l-tryptophan accumulation and l-valine accumulation. The strains were cultivated in biological triplicates (quadruplicates for the plasmid-harboring strains) in 50 ml CGXII media supplemented with 111 mM glucose in 500 ml baffled shake flasks. The cultivation started with an OD_600_ = 0.8, and 200 µM IPTG and 25 µg/ml kanamycin were added to the plasmid-harboring strains. The mean values are shown as diamonds, and the standard deviations are shown as lines

The growth performance as well as l-tryptophan and l-valine accumulation were analyzed in a shake flask experiment (Fig. 4). Compared with the parental strain, the deletion mutant WT TRP^+++^ Δ*trpP* had a lower growth rate and an 8% lower maximum OD_600_ (Fig. 4A). WT TRP^+++^ pPREx2-*trpP* (*trpP* overexpression) grew slightly slower than WT TRP^+++^ pPREx2 (empty plasmid) (Fig. 4B). Interestingly, both TrpP deletion and overexpression led to increased l-tryptophan accumulation (Fig. 4). Thus, TrpP is most likely not a typical l-tryptophan importer or exporter. Furthermore, *trpP* deletion reduced the formation of the byproduct valine (Fig. 4A), whereas *trp* overexpression increased valine formation more than 3-fold (Fig. 4B). Valine is usually formed when pyruvate accumulates, but none of the mutations in WT TRP^+++^ suggest pyruvate accumulation. Thus, the reason for the l-valine accumulation of WT TRP^+++^ is currently unknown. In summary, altering of *trpP* expression levels appears to be beneficial for l-tryptophan production but not due to a change in l-tryptophan transport.

### Transcriptome comparison of WT Δ *trpP* and WT

**Fig. 5:**
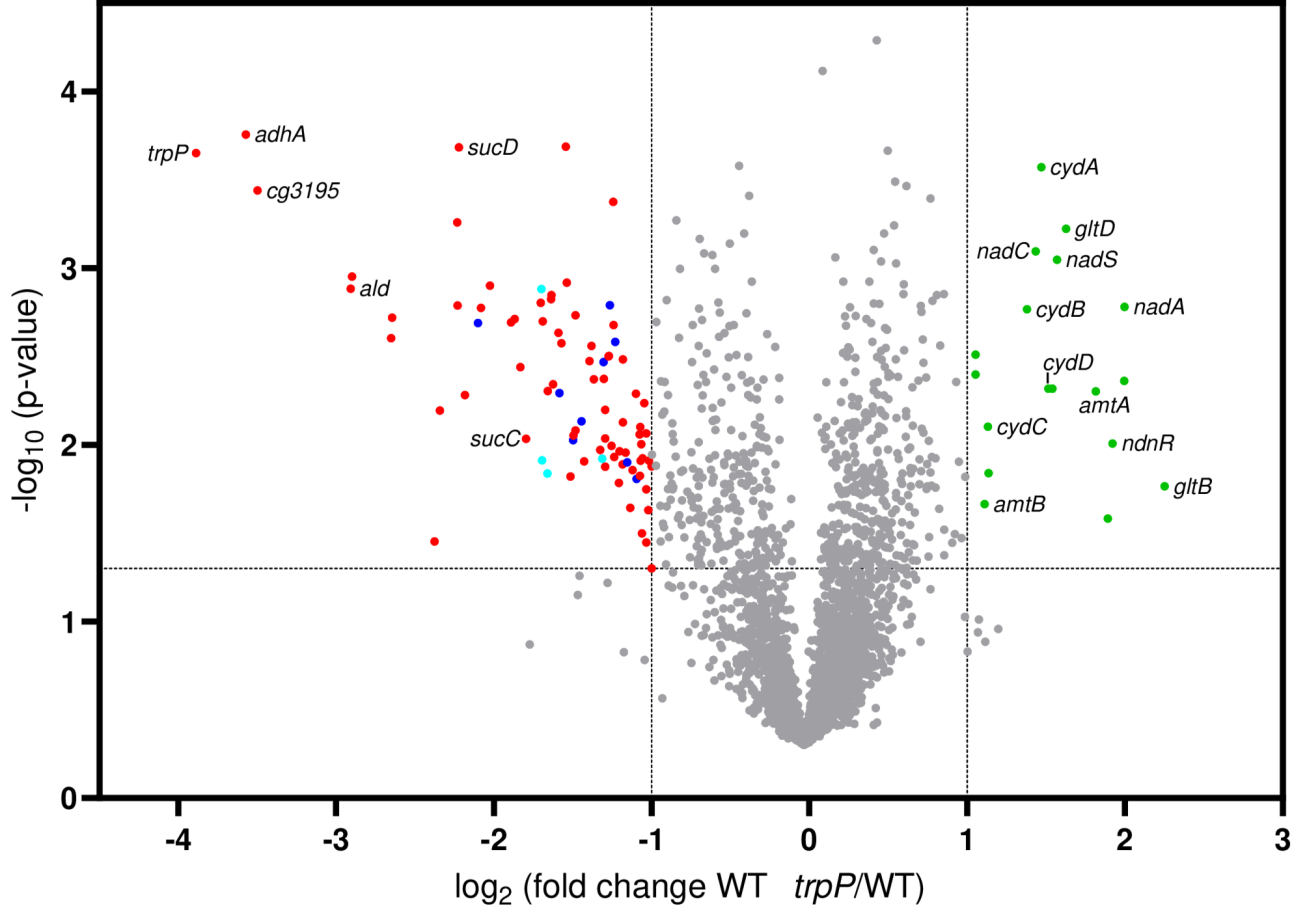
Volcano plot of the transcriptome analysis of WT Δ*trpP* vs. WT. The strains were cultivated in defined CGXII media with 111 mM glucose as carbon source and harvested in the exponential growth phase (OD_600_ = 5). The colored dots represent genes whose expression was regulated at least 2-fold in three out of four experiments, *p* < 0.05, signal to noise ratio > 2. The gray dots represent genes that did not match these criteria. Blue dots: genes involved in *myo*-inositol transport and metabolism. Cyan dots: genes involved in citrate transport and metabolism. Red and green dots: other up- or downregulated genes

The characterization of the *trpP* deletion and overexpression strains regarding l-tryptophan production argued against TrpP being an l-tryptophan transporter. Thus, we wanted to test the second hypothesis that TrpP might have a regulatory function. For this, we performed a transcriptome comparison of WT Δ*trpP* and WT in CGXII with 111 mM glucose as carbon source (Fig. 5, Table S3). Please note that growth of these two strains was slightly different (Fig. 1B), thus we might have detected altered genes as secondary effects due to the growth defect. Overall, in WT Δ*trpP*, 82 genes presented at least a 2-fold reduction in transcription and 18 genes presented at least a 2-fold increase in transcription (3 out of 4 experiments, *p* < 0.05, signal to noise ratio > 2) (Fig. 5, Table S3). *trpP* was the most downregulated gene, confirming its deletion. As [Fe-S] clusters will be important later in this manuscript, we mention here when proteins contain [Fe-S] clusters.

Among the genes whose expression was most strongly upregulated (2.7-4.0-fold) was the *ndnRnadACS* operon (cg1214-cg1218), which is required for *de novo* NAD biosynthesis [45]. The operon is repressed at the transcriptional level by NdnR and WhcA, a WhiB-like regulator containing an [4Fe-4S] cluster [46, 47]. The genes that were upregulated the most in WT Δ*trpP* (3.1-4.8-fold) were *gltBD* (cg0229-cg0230), encoding glutamine 2-oxoglutarate aminotransferase (GOGAT). Notably, GltD harbors a [4Fe-4S] cluster. In *C. glutamicum*, GOGAT, together with glutamine synthetase (GS, encoded by *glnA*, cg2429), represents one of the two main pathways for ammonium assimilation. A further operon of interest that was upregulated 2.6-fold in WT Δ*trpP* was the *cydABCD* operon (cg1298-1301), encoding cytochrome *bd* oxidase (*cydAB*) and a transporter required for the cytochrome *bd* oxidase assembly (*cydCD*). *C. glutamicum* has two terminal oxidases, the cytochrome *bd* oxidase and the cytochrome *aa*_3_ oxidase, the latter of which forms a supercomplex with the cytochrome *bc*_1_ complex [48, 49]. The supercomplex is more efficient in contributing to the proton motive force by pumping protons, whereas the cytochrome *bd* oxidase is preferred under low-oxygen conditions because of its higher oxygen affinity [50, 51].

Among the downregulated genes were many involved in uptake and metabolism of various carbon sources, such as ethanol (*ald* and *adhA*), *myo-*inositol (blue dots), citrate (cyan dots), protocatechuate, propionate, gluconate, vanillate, and 4-cresol. These are often under control of GlxR, the global regulator that responds to the cAMP concentration in the cell. This finding is in line with the reduced growth of WT Δ*trpP* with various carbon sources (Fig. S1) and suggests that the cAMP level might be altered in WT Δ*trpP*.

Overall, *trpP* deletion in *C. glutamicum* affected the transcription of quite many genes, several of which are related to impaired growth of the mutant. However, there was no obvious indication of a specific regulatory function of TrpP, so further studies were needed.

### Evolution of C1* ΔTRP Δ *trpP* toward better growth reveals unexpected target

As previous experiments did not reveal a clear reason why *trpP* deletion impairs growth, we used ALE to select for Δ*trpP* strains with faster growth as an additional approach to understand the Δ*trpP* defect [29, 30]. Please note that the ALE was performed with strain C1* ΔTRP Δ*trpP* and not with WT ΔTRP Δ*trpP*. In the CoNoS project, we worked in parallel with the WT and the chassis strain C1*. C1* is a variant of the WT with a 13.4% reduced genome [21], and the C1*-based l-tryptophan auxotrophic strain was available earlier than the WT-based strain. C1* ΔTRP Δ*trpP* has an even lower growth rate than WT ΔTRP Δ*trpP* (Fig. S5A); consequently, there was slightly greater selective pressure on the strain to grow faster. In CGXII glucose media supplemented with 0.5 g L^− 1^l-tryptophan, C1* ΔTRP Δ*trpP* had a growth rate corresponding to approximately 50% of that of the parental strain C1* (Fig. S5B). As we later confirmed the effects of the evolved mutations in the WT background, it was not necessary to repeat ALE with a WT-based strain.

**Fig. 6:**
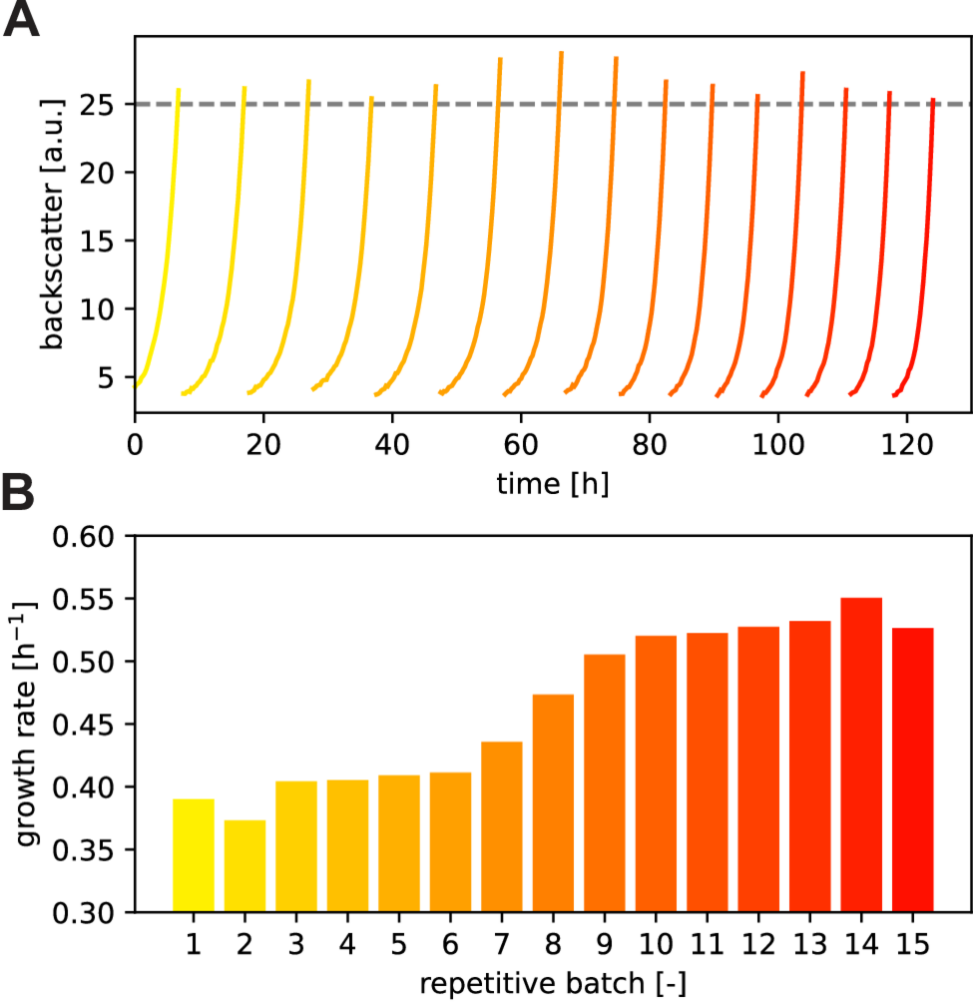
ALE with C1* ΔTRP Δ*trpP* via repetitive batch cultures. The fully automated experiment was performed on a Mini Pilot Plant and each batch was started with freshly stored CGXII medium (111 mM glucose, 0.5 g L^− 1^l-tryptophan). After the predefined backscatter of BS = 25 was reached, the next batch was inoculated with cells from the previous batch to an OD_600_ of 0.5. (**A**) Online backscatter measurements from a representative ALE from three independent replicates. (**B**) Evolution of the maximum specific growth rates along the repetitive batches

ALE was performed in triplicate with l-tryptophan supplementation. The growth curves of one representative evolution experiment over sixteen cycles are shown in Fig. 6A. During this time, the growth rate increased by at least 25% in all three replicates (Fig. 6B). From each replicate, single clones were isolated from the last batch and tested again for growth performance in biological duplicates. For all three replicates, the growth rate of the selected clones increased significantly to at least 0.49 h^− 1^ (Fig. S5C), suggesting permanent genomic changes. To identify the mutations responsible for improved growth, we isolated genomic DNA from the evolved cultures and sequenced it via whole-genome sequencing. Notably, all three clones were found to carry mutations in the coding region of the same gene, *sufR* (cg1765), but at different codons: L25P (evo1), R77G (evo2) and Q193* (evo3) (Tab. S4). Besides some silent mutations, these were the only mutations we found in all three strains with a frequency of > 90%. SufR (228 amino acid residues) is a transcriptional regulator that functions as a repressor of the *sufBDCSUT* operon (cg1764-cg1759, referred to as the *suf* operon) [52]. The protein is composed of an ArsR-type HTH motif in the N-terminal half and a [4Fe-4S] cluster binding domain in the C-terminal half. The *suf* operon encodes proteins for Fe-S cluster biogenesis and repair [53–55]. *C. glutamicum* does not possess any other system for [Fe-S] cluster formation, such as the iron–sulfur cluster (ISC) system or the nitrogen fixation (NIF) system. The amino acid residues L25 and R77 are highly conserved in SufR, whereas Q193* leads to a shortened version of the protein (Fig. S6). We assume that all the identified mutations lead to a loss of SufR function, causing derepression of the *suf* operon.

### Reconstruction of the evolved mutations and confirmation of their growth-improving effects

**Fig. 7:**
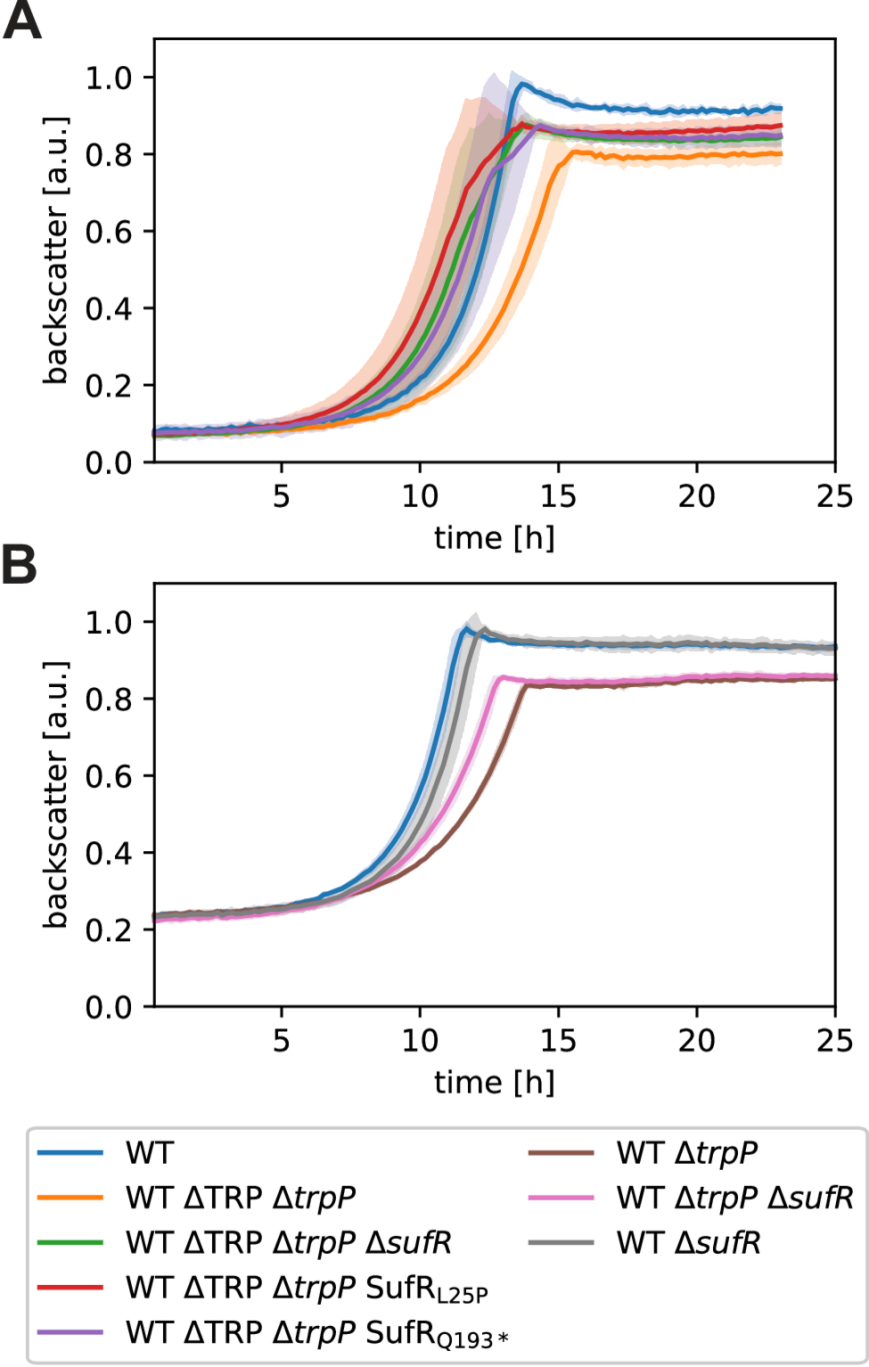
Growth of *C. glutamicum* strains with reengineered mutations that evolved during ALE. (**A**) Cultivation of WT ΔTRP-derived strains. (**B**) Cultivation of WT derived strains. All cultures were cultured in CGXII media supplemented with 111 mM d-glucose and 0.5 mM l-tryptophan for auxotrophic strains at 30 °C, 1400 rpm, and 85% humidity. The backscatter data were normalized on the basis of the maximum value recorded for each WT in the corresponding experiment. The mean values of biological triplicates are shown as lines, and standard deviations are shown as shaded areas

To test whether the mutations we found were indeed responsible for the improved growth of the evolved strains, we introduced two of them into WT ΔTRP Δ*trpP*, yielding WT ΔTRP Δ*trpP* SufR_L25P_ and WT ΔTRP Δ*trpP* SufR_Q193*_. Furthermore, we constructed strain WT ΔTRP Δ*trpP* Δ*sufR* with a chromosomal deletion of the *sufR* gene while leaving the two promoters in front of *sufR* and *sufB* intact. All three *sufR-*affected strains grew faster than the parental strain WT ΔTRP Δ*trpP* (Fig. 7A), which confirms the growth-promoting effect of SufR inactivity in the Δ*trpP* strain. To confirm that this effect is similar in a nonauxotrophic *trpP* deletion strain, we constructed strain WT Δ*trpP* Δ*sufR*. As expected, *sufR* deletion led to partial complementation of the growth-retarding effect of Δ*trpP* (Fig. 7B). In contrast, deletion of *sufR* in the WT background did not lead to any improvement in growth under the conditions tested (Fig. 7B). In summary, we confirmed that the loss of SufR function can partially compensate for the negative effect of *trpP* deletion.

### Regulation of the *suf* operon by SufR

SufR is a transcriptional repressor of the *suf* operon [52]. To date, this interaction has not been characterized in detail in *C. glutamicum*, but several studies of SufR function in other organisms, such as *Mycobacterium tuberculosis* [56] or *Streptomyces avermitilis* [57], are available. The *suf* operon is also regulated by OxyR in response to hydrogen peroxide stress [58]. SufR is a homodimer, and each dimer carries a [4Fe-4S] cluster in the C-terminal region, which is used to sense the intracellular [4Fe-4S]-cluster status [57]. The [4Fe-4S] cluster coordinating residues in *C. glutamicum* SufR are presumably at positions 171, 175, 188, and 216 (Fig. S6). SufR acts as a repressor of the *suf* operon when the [4Fe-4S] clusters in SufR are intact. Once the [4Fe-4S] clusters are oxidized or lost, SufR is released from the promoter, and the *suf* operon is transcribed, which should increase [Fe-S] cluster formation and repair.

**Fig. 8:**
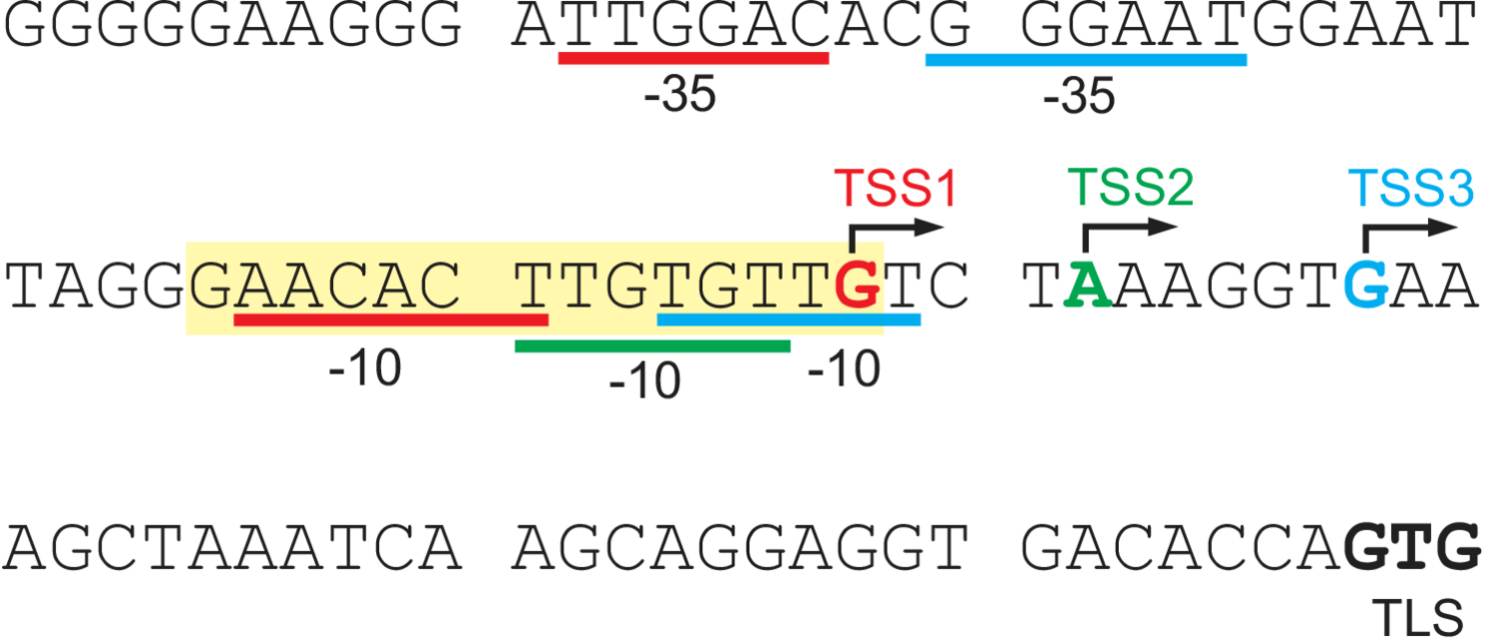
Transcriptional organization of *C. glutamicum sufR*. TSS1, TSS2 and TSS3 indicate the three transcriptional start sites. The corresponding − 10 and − 35 regions (if known) are underlined in the respective color. The yellow shaded region corresponds to the SufR binding site of *Streptomyces avermilitis.* The translational start site (TLS) of SufR is written in bold black letters. Data according to [34, 52, 59]

In the literature, three transcriptional start sites are known for SufR in *C. glutamicum*. The housekeeping and presumably most important promoter is TSS1 [34, 52], a less important promoter is TSS2 [34], and TSS3 is a SigM-dependent promoter [52] (Fig. 8). Furthermore, there is an additional promoter within the *sufR* coding region, but this seems to be of minor importance for the transcription of the *suf* operon [34].

SufR of *S. avermitilis* binds to an inverted repeat (CAAC-N_6_-GTTG) in the *sufR* promoter, where the first part overlaps with the − 10 region (AACAAT) and the last “G” is identical to the transcriptional start site (TSS) of SufR [57]. This motif (GAAC-N_6_-GTTG) and TSS1 are almost perfectly conserved in *C. glutamicum* (Fig. 8).

To test whether TSS1 is the major TSS of SufR, we performed a promoter activity assay. For this purpose, we constructed the plasmid pJC1-P_sufR_-venus, in which the *sufR* promoter is fused to the coding region for the fluorescent protein mVenus at the start codon of SufR, thus retaining the native ribosome binding site. This allowed direct measurement of promoter activity by fluorescence intensity. In the plasmid pJC1-P_sufR_-venus, we then mutated the two − 10 regions belonging to TSS1 and TSS2, resulting in the plasmids pJC1-P_sufR_-venus-Mut_TSS1_ and pJC1-P_sufR_-venus- Mut_TSS2_ (Fig. S7A). As both − 10-regions are located within the predicted SufR binding site, SufR binding could also be influenced by these mutations. All plasmids were used to transform *C. glutamicum* WT. The resulting strains were analyzed via a Biolector experiment in which growth and fluorescence were monitored (Fig. S7B and S7C). None of the plasmids influenced growth, which allowed easy analysis of the fluorescence (Fig. S7B). The fluorescence of the strain WT pJC1-P_sufR_-venus was much greater than that of the control strain WT without plasmid, confirming the functionality of the fusion construct (Fig. S7C). The fluorescence of the strain with pJC1-P_sufR_-venus-Mut_TSS2_ was similar to that of the strain with pJC1-P_sufR_-venus, which suggests that TSS2 is only of minor importance for SUF transcription. In contrast, the strain with pJC1-P_sufR_-venus-Mut_TSS1_ had much lower fluorescence than the strain with pJC1-P_sufR_-venus; thus, the mutation of this − 10 region significantly reduced transcription (Fig. S7C). As we did not observe increased fluorescence upon mutation, we assume that mutation of the SufR binding site was only of minor importance here. In summary, TSS1 appears to be the major TSS of the *suf* operon.

### Increased *suf* operon expression is responsible for the growth-promoting effect of SufR inactivation in a *trpP* deletion mutant

**Fig. 9:**
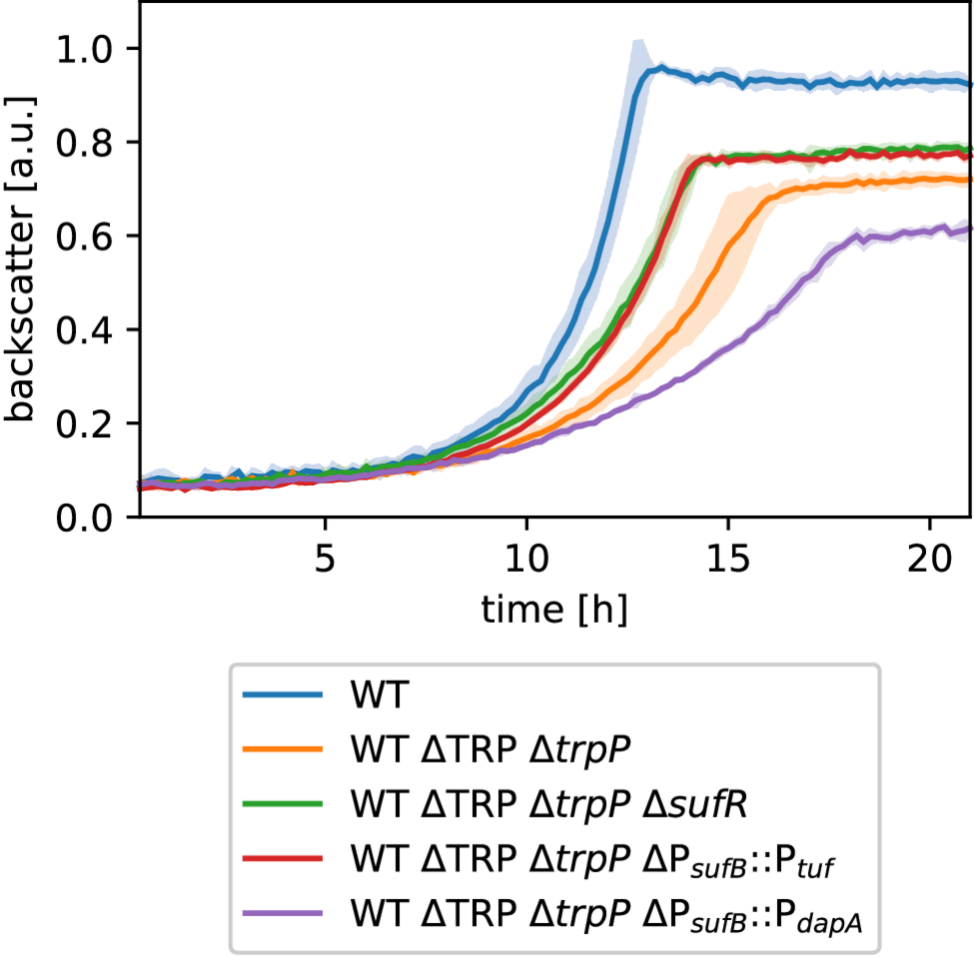
Testing different promoters in front of *sufB* in WT ΔTRP Δ*trpP* Δ*sufR*. All cultures were cultured in CGXII media supplemented with 111 mM d-glucose and 0.5 mM l-tryptophan for auxotrophic strains at 30 °C, 1400 rpm, and 85% humidity. The backscatter data were normalized on the basis of the maximum value recorded for each WT in the corresponding experiment. The mean values are shown as lines, and the standard deviations as shaded areas

We showed above that inactivation or deletion of *sufR* partially complemented the decrease in growth caused by *trpP* deletion. Repression of the *suf* operon is likely the main SufR function, as other potential target genes are not known. Thus, we wanted to determine whether increased expression of the *suf* operon is indeed responsible for the growth-promoting effect of Δ*sufR* in WT Δ*trpP*. Since plasmid-based expression of the *suf* operon was problematic (data not shown), we exchanged the native promoter of *sufB* (P_*sufB*_) in WT ΔTRP Δ*trpP* with two other *C. glutamicum* promoters, the strong P_*tuf*_ promoter and the weak P_*dapA*_ promoter [60], and analyzed growth (Fig. 9). The strain with P_*tuf*_ in front of *sufB* grew better than the parental strain WT ΔTRP Δ*trpP*, with a growth rate similar to that of WT ΔTRP Δ*trpP* Δ*sufR*, which means that strong *suf* operon expression is beneficial under these conditions. WT ΔTRP Δ*trpP* ΔP_*sufB*_::P_*dapA*_ grew even slower than WT ΔTRP Δ*trpP*, and to only 50% maximal backscatter compared to WT, suggesting that reduced *suf* operon expression reinforces the negative effect of *trpP* deletion. The observed growth suggested a positive correlation between an increased expression level of the *suf* operon and strain growth. Thus, our results confirm that increased *suf* operon expression is presumably the main effect of *sufR* deletion or inactivation.

### Influence of TrpP and SufR on tryptophan production

**Fig. 10:**
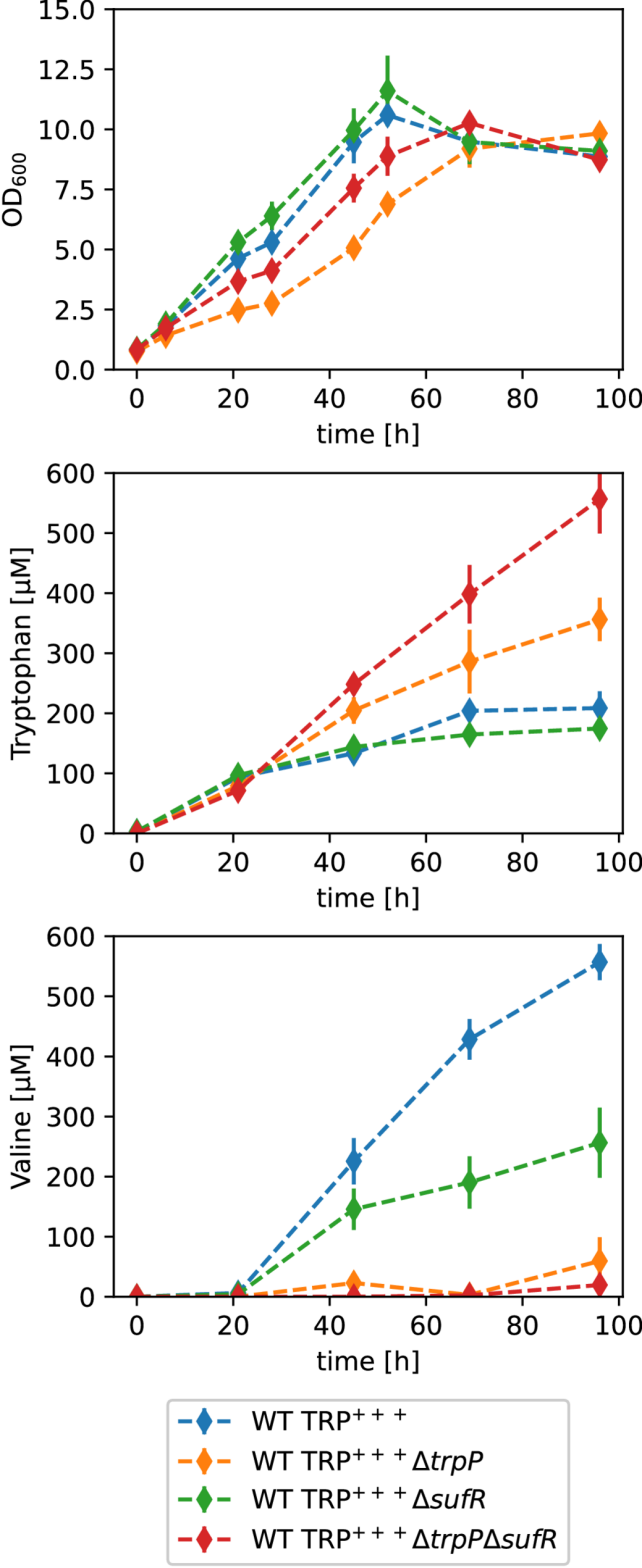
Characterization of the influence of *sufR* on amino acid formation in *C. glutamicum*. Cultivation of WT TRP^+++^ derived *trpP*, *sufR* and double deletion strains and analysis regarding their growth, their l-tryptophan accumulation, and their l-valine accumulation. The strains were cultivated in 50 ml CGXII medium with 111 mM d-glucose in 500 ml baffled shake flasks. The cultivation was started with an OD_600_ = 0.8. l-tryptophan and l-valine concentrations were determined by HPLC. Mean values of biological triplicates are shown as diamonds, and standard deviations as lines

Above, we showed that *trpP* deletion led to increased l-tryptophan production, possibly because it reduced the formation of the byproduct l-valine (Fig. 4). As *sufR* deletion partially complemented the growth defect of the Δ*trpP* strain, we wanted to determine whether this deletion also has an influence on l-tryptophan production. We used the same l-tryptophan-producing strain as before (WT TRP^+++^) and constructed two additional strains, WT TRP^+++^Δ*sufR* and WT TRP^+++^Δ*trpP* Δ*sufR*, and analyzed growth, l-tryptophan and l-valine production in a shake flask experiment (Fig. 10).

As previously observed for the WT (Fig. 7B), *trpP* deletion decreased the growth rate of WT TRP^+++^ (Fig. 10). Single deletion of *sufR* slightly increased the growth rate of WT TRP^+++^, whereas WT TRP^+++^ Δ*trpP* Δ*sufR* grew slower than WT TRP^+++^ but significantly faster than WT TRP^+++^ Δ*trpP* (Fig. 10). l-Tryptophan remained unchanged upon deletion of *sufR* and increased by 71% upon deletion of *trpP* in comparison to WT TRP^+++^ (Fig. 10). Compared with the *trpP* single deletion, the *trpP sufR* double deletion led to even faster and stronger l-tryptophan production, with approximately 3-fold more l-tryptophan than WT TRP^+++^ (Fig. 10). SufR deletion alone decreased l-valine formation by more than 50% (Fig. 10). *trpP* deletion alone or in combination with *sufR* almost completely abolished l-valine production (Fig. 10). In summary, the deletion of *trpP* increased l-tryptophan production and reduced by-product formation. Additional deletion of *sufR* increased l-tryptophan production further, but did not have an additional positive effect on byproduct formation.

## Discussion

In our study, we wanted to elucidate the cause of the growth limitation of the auxotrophic strain WT ΔTRP Δ*trpP* and to investigate the potential role of TrpP in l-tryptophan transport, metabolism or regulation. A growth experiment with WT ΔTRP and WT Δ*trpP* revealed that the growth defect was caused solely by *trpP* deletion and was thus independent of the l-tryptophan auxotrophy caused by deletion of the *trp* operon. On the basis of the results of the l-tryptophan production experiment with *trpP* deletion and overexpression, we can exclude a clear l-tryptophan importer or exporter function of TrpP. Transcriptome analysis, ALE and complementation experiments suggested that *trpP* deletion has a negative effect on the formation/repair of [Fe-S] clusters, which can be partially complemented by overexpression of the *suf* operon through inactivation of the repressor SufR. Finally, we discovered that *trpP* deletion increased l-tryptophan production in a model producer, which was even further enhanced by additional deletion of *sufR*.

The transcriptome comparison of the *trpP* deletion strain with the wild-type strain as well as the ALE experiment suggested that *trpP* deletion influences the formation and repair of [Fe-S] clusters. We estimate that *C. glutamicum* has approximately 30–50 [Fe-S] cluster containing proteins that are involved in various cellular processes, such as central carbon metabolism (aconitase, *acn*, cg1737; succinate: menaquinone oxidoreductase, *sdhB*, cg0447), the respiratory chain (Rieske iron‒sulfur protein, *qcrA*, cg2404), amino acid biosynthesis (isopropylmalate isomerase, *leuC*, cg1487; dihydroxyacid dehydratase, *ilvD*, cg1432), ammonium assimilation (*gltD*, cg0230), and NAD synthesis (*nadA*, cg1216), as well as signaling and regulation (*arnR*, cg1340; *sufR*, cg1765, WhiB-like proteins, cg0337, cg0695, cg0850, cg0878).

*C. glutamicum* has two pathways for ammonium assimilation. In the presence of sufficient ammonium supply, assimilation via glutamate dehydrogenase (*gdh*, cg2280) is usually favored because the GS/GOGAT (*glnA* (cg2429), *gltBD* (cg0229-cg0230)) pathway requires ATP for ammonium fixation and is thus more expensive for the cell [61, 62]. Only in the case of nitrogen limitation is GOGAT upregulated. Regulation at the transcriptional level is performed by AmtR (Repressor) and possibly GlxR [63–66]. In line with potential nitrogen limitation, the ammonium transporter genes *amtA* (cg1785) and *amtB* (cg2261) were upregulated more than twofold in WT Δ*trpP*. In summary, these data suggested reduced ammonium availability in WT Δ*trpP*. However, in WT Δ*trpP*, only parts of the AmtR regulon were altered, and the CGXII medium used contained much more ammonium than was required for cell growth. Thus, the observed effect presumably has reasons other than ammonium limitation [67].

*C. glutamicum* has two terminal oxidases, the cytochrome *bd* oxidase and the cytochrome *aa*_3_ oxidase, the latter of which forms a supercomplex with the cytochrome *bc*_1_ complex [48, 49, 68]. The supercomplex is more efficient in contributing to the proton motive force by pumping protons, whereas cytochrome *bd* oxidase is preferred under low-oxygen conditions because of its higher oxygen affinity [50, 51]. The *cydABCD* operon is regulated by OxyR, which senses hydrogen peroxide stress [58]. Both its upregulation and its deletion reduce the growth efficiency, i.e., the final OD [51]. This is assumed to be due to the threefold lower efficiency of cytochrome *bd* oxidase than that of the major cytochrome *bc*_1_-*aa*_3_ supercomplex in building up proton-motive force [51]. Cytochrome *bd* oxidase was suggested to be particularly relevant under low-oxygen conditions because of its assumed high oxygen affinity [50], and it was shown to be essential under copper-deficient conditions, as cytochrome *aa*_3_ oxidase activity is dependent on copper, whereas cytochrome *bd* oxidase activity is not [69]. In fact, a *cydAB* deletion mutant was unable to grow under copper deprivation [69]. Another difference between cytochrome *bd* oxidase and the cytochrome *bc*_1_-*aa*_3_ supercomplex is the presence of a periplasmic [2Fe-2S] cluster in the Rieske iron sulfur protein, whereas the *bd* oxidase does not harbor an [Fe-S] cluster and could therefore be important under conditions where the formation of [Fe-S] clusters is hampered. As we can most likely exclude oxygen limitation and copper depletion in our experiments, the increased transcription of *cydABCD* also suggests a reduced formation or repair of [Fe-S] clusters.

The increased expression of *ndnRnadACS* (cg1214-cg1218) was presumably caused by reduced repression by NdnR, WhcA or both. NdnR is a transcriptional regulator belonging to the NrtR family. NadAC encodes the machinery for *de novo* NAD biosynthesis, with NadA containing a [4Fe-4 S] cluster [45]. NadS is a NifS-type cysteine desulfurase involved in [4Fe-4S] cluster assembly [45]. The binding of NdnR to the promoter is enhanced by NAD, which acts as a corepressor [45, 47]; thus, increased expression of this cluster could result from reduced NAD availability. WhcA contains a [4Fe-4S] cluster and represses genes involved in the oxidative stress response (Table S5) [46]. It directly interacts with SpiA (cg1064), which presumably influences the regulatory activity of WhcA [46].

WhcA is a WhiB-like (Wbl) protein. Wbl proteins are small proteins of approximately 40–140 amino acids that are exclusively present in Actinobacteria [70, 71]. Each Wbl protein contains an O_2_ and NO-sensitive [4Fe-4S] cluster that is coordinated by four cysteine residues [70]. They control transcription either via direct binding to DNA or via interaction with a mediating protein [70]. In addition to WhcA, *C. glutamicum* possesses three other Wbl proteins that have been characterized to some extent: WhcB (Cg0695), WhcD (Cg0850), and WhcE (Cg0878). Although all Wbl proteins possess a [4Fe-4S] cluster, in our transcriptome analysis we observed alterations only for genes regulated by WhcA, but not for genes known to be regulated by the other Wbl proteins WhcB, WhcE or WhcE (please see Table S5). This is not too surprising, because the majority of genes is regulated by more than one regulator to respond to different input signals. Thus, e.g. under different growth conditions we might also see changes for the genes regulated by WhcB, WhcE or WhcE upon *trpP* deletion. In summary, we clearly saw that TrpP presence influenced genes regulated by WhcA, presumably by affecting [Fe-S] cluster formation and repair. For further details regarding Wbl proteins, please see Table S5 and the supplemental discussion.

The l-tryptophan production experiments demonstrated that both *trpP* deletion and *trpP* overexpression had a positive effect on production. Obviously, there must be different mechanisms how the l-tryptophan production is influenced by TrpP availability. Deletion of *trpP* almost completely abolished the accumulation of the byproduct l-valine. l-valine is formed from pyruvate in four steps via acetolactate, dihydroxyisovalerate and 2-ketoisovalerate. The enzymes involved are acetohydroxy acid synthase (AHAS), acetohydroxy acid isomeroreductase (AHAIR), dihydroxyacid dehydratase (DHAD) and transaminase B (TA). As mentioned above, the reason for the l-valine accumulation of WT TRP^+++^ is currently unknown. DHAD, encoded by *ilvD* (cg1432), contains a [4Fe-4S] cluster (IPR004404) [39]. The *trpP* deletion, which presumably reduces [Fe-S] cluster formation, could reduce l-valine production by decreasing the availability of active DHAD. The increase in l-tryptophan in the same experiment might be a secondary effect of the reduced byproduct formation. Alternatively, this could be caused by increased precursor (PEP) availability due to reduced flux through the TCA cycle, which is caused by reduced activity of the [4Fe-4S] cluster containing aconitase [72]. Interestingly, *sufR* deletion also reduced the formation of l-valine, although it should have the opposite effect as *trpP* deletion by increasing [Fe-S] cluster formation. Thus, we assume that l-valine formation is influenced by TrpP and SufR via different processes. Interestingly, both l-tryptophan and l-valine showed an increased accumulation upon *trpP* overexpression. Here, a different mechanism than the one mentioned above must be relevant. For example, the increased l-valine could be a consequence of increased pyruvate accumulation during l-tryptophan biosynthesis. One molecule of pyruvate is formed per molecule of l-tryptophan by anthranilate synthase and another one could be formed from glyceraldehyde-3-phosphate, which is a product of l-tryptophan synthase [73]. Deletion of *sufR* in WT TRP^+++^ Δ*trpP* led to a further increase in l-tryptophan production (Fig. 10). Production of l-tryptophan requires the cofactor pyridoxal 5’-phosphate (PLP). During synthesis of PLP, hydrogen peroxide is formed [74], which may increase oxidative stress within the cell and thereby the requirement for [Fe-S] clusters. Additional deletion of *sufR* should increase [Fe-S] cluster assembly and thereby help to reduce oxidative stress. Overall, the positive effect of combined *trpP sufR* deletion on l-tryptophan production was relatively strong and is thus worthy of further study.

When our data were analyzed, we asked how *trpP* deletion could influence the formation and repair of [Fe-S] clusters. We can exclude the possibility that this phenomenon is caused by problems with iron acquisition because we do not observe typical transcriptional changes upon iron starvation, e.g., in the DtxR or RipA regulons [75]. Problems with sulfur metabolism are also unlikely because we do not observe any expression changes in genes involved in sulfur metabolism upstream of the [Fe-S] cluster assembly [76, 77]. Another option is that TrpP influences [Fe-S] cluster assembly, repair or degradation. The deletion of *trpP* had no direct effect on *suf* operon transcription, so TrpP possibly affects these proteins at the posttranscriptional level, possibly through direct protein‒protein interactions with components of the [Fe–S] cluster assembly machinery. The reduced availability of intact [Fe-S] clusters has severe effects on the whole cell. Central carbon metabolism is likely reduced because [Fe-S]-containing proteins such as aconitase are involved in the TCA cycle. This could explain the reduced growth rate and final biomass of WT Δ*trpP* in comparison with those of the WT.

The similarity of TrpP to the SdpI protein of *B. subtilis* suggested a regulatory function for TrpP [18, 19]. However, we must consider that (i) TrpP is only half of the length of SdpI, (ii) the other parts of the signal cascade in *B. subtilis* (SdpR and SdpC) are missing in *C. glutamicum*, and (iii) SdpI is thus far the only characterized representative of this protein family and may represent only a small part of possible functions. SdpI contains six transmembrane helices (Fig. 3B). The first three form the SdpC-binding domain, and the helices four-six form the SdpR-binding domain. The alignment of SdpI and TrpP revealed that TrpP is homologous to the SdpR-binding domain of SdpI, with an additional C-terminal extension that is absent from SdpI (Fig. 3). The identity between SdpI and TrpP in the aligned region is 26.76%. This suggests that TrpP also directly interacts with another protein. Currently, there is no obvious candidate for the interacting protein, as there is no homolog of SdpR in *C. glutamicum*. The C-terminal extension might influence TrpP activity, e.g., by binding to a possible ligand.

## Conclusion

**Fig. 11:**
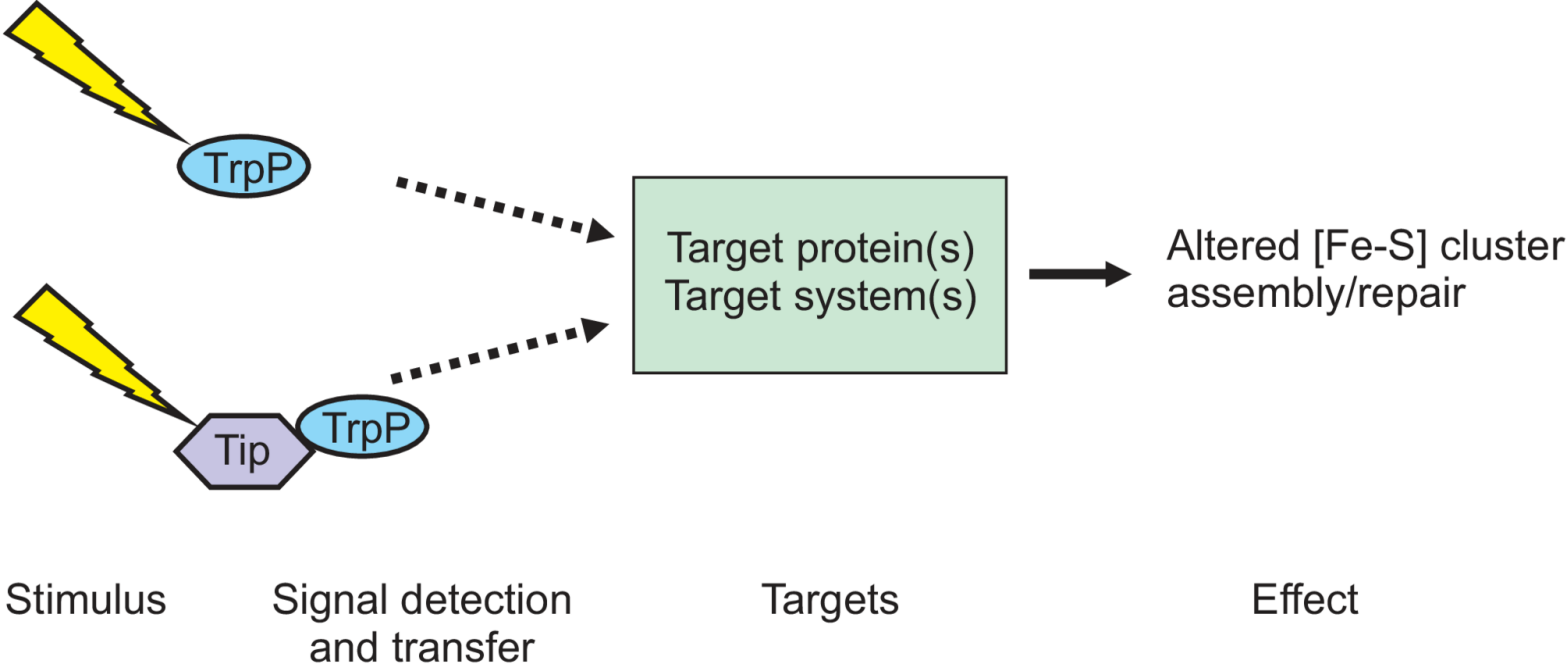
Schematic model of a putative signal transduction pathway involving TrpP. Yellow flash: stimulus, Tip: TrpP interacting protein

In our study, we wanted to investigate the potential role of TrpP in l-tryptophan transport, synthesis or regulation and to elucidate the cause of the growth limitation of a Δ*trpP* mutant. According to our data, it is rather unlikely that TrpP is truly an l-tryptophan transporter. These findings point more toward a possible regulatory or completely unrelated function. In this work, we found an interesting link between TrpP and the *suf* operon, the only [Fe-S]-cluster assembly machinery of *C. glutamicum*. TrpP seems to influence the formation and repair of [Fe-S] clusters. Thus, the reduced growth of WT Δ*trpP* is presumably caused by the reduced activity of [Fe-S]-cluster-containing enzymes involved in central metabolism, such as aconitase or succinate: menaquinone oxidoreductase. However, SufR deletion caused increased *suf* operon expression, which only partially complemented the growth reduction caused by *trpP* deletion. Thus, further effects of *trpP* deletion may be discovered. Based on our research, we developed a very basic schematic model of TrpP function (Fig. 11). A so far unknown stimulus is sensed either directly by TrpP or by another protein that transfers the signal to TrpP. The signal is then further transferred to the target system(s) or target protein(s) by a so far unknown mechanism, with the effect that Fe-S cluster assembly and/or repair is altered (Fig. 11). As the TrpP domain organization is so different from all known proteins involved in signal transduction processes, it might function with an entirely new mechanism. To better understand TrpP function, it would be interesting to search for potential interacting proteins. Furthermore, the importance of the C-terminal extension could be elucidated by complementation studies with truncated protein variants. Finally, we showed how *trpP* and *sufR* deletion increased l-tryptophan production by reducing byproduct formation.

## Electronic supplementary material

Below is the link to the electronic supplementary material.

Supplementary Material 1

## Data Availability

The genome sequencing data generated during this study has been deposited in the European Nucleotide Archive (ENA) at EMBL-EBI under accession number PRJEB76054 (https://www.ebi.ac.uk/ena/browser/view/PRJEB76054). The full microarray datasets of this study have been deposited in the NCBI Gene Expression Omnibus (https://www.ncbi.nlm.nih.gov/geo/query/acc.cgi) and can be found under the GEO accession number GSE272128. All other data generated or analyzed during this study are included in this published article and its supplementary information files. Strains and plasmids generated during this study are available from the corresponding author upon request.
