# Supplementary material for: ALE reveals a surprising link between [Fe-S] cluster formation, tryptophan biosynthesis and the potential regulatory protein TrpP in Corynebacterium glutamicum
Source: BMC Microbiol. 2025 Apr 14;25:214. doi: 10.1186/s12866-025-03939-z (PMC11995493; doi:10.1186/s12866-025-03939-z)
Supplement: Supplementary file 1 — Supplementary Material 1 [file 12866_2025_3939_MOESM1_ESM.pdf]

Supplement to

ALE reveals a surprising link between [Fe-S] cluster formation, tryptophan biosynthesis and the potential regulatory protein TrpP in *Corynebacterium glutamicum*

Zuchowski, Rico[1], Schito, Simone[1], Mack, Christina[1], Wirtz, Astrid[1], Bott, Michael[1], Polen, Tino[1], Noack, Stephan[1], Baumgart, Meike[1#]

[1]Institut für Bio- und Geowissenschaften, IBG-1: Biotechnologie, Forschungszentrum Jülich, Jülich, Germany

#Address correspondence to m.baumgart@fz-juelich.de

Keywords: tryptophan, *suf* operon, SufR, *Corynebacterium glutamicum*, auxotrophy, [Fe-S] cluster, *trp* operon, adaptive laboratory evolution, ALE

Supplemental Discussion

In the following, we would like to discuss the potential effects of altered iron-sulfur-cluster availability on WhiB-like protein (Wbl) function, because they potentiate the effects of altered [Fe-S] cluster availability. Wbls are small proteins of about 40-140 amino acids and exclusively present in Actinobacteria (1, 2). Each Wbl contains an O_2 and NO-sensitive [4Fe-4S] cluster that is coordinated by four cysteine residues (1). They control transcription either via direct binding to DNA via interaction with a mediating protein (1). *C. glutamicum* has four Wbl proteins as well as a potential WhiA-homolog (cg1792, potential interaction with WhcD), which have been characterized to some extent (Table S5). WhcA (WhiB4) is not essential and potentially involved in transcriptional regulation of NADH oxidase, alcohol dehydrogenase, quinone reductase and cysteine desulfurase (3) (Table S5). According to the current model, under normal growth conditions, SpiA (Cg1068) reduces Apo-WhcA (S-S) to its holo-form ([4Fe-4S]). In this process, WhcA receives its [4Fe-4S] cluster, gains the ability to bind DNA and thus represses genes involved in oxidative stress response (4). Activity of WhcA is regulated at three levels: i) expression of *whcA* and *spiA,* ii) WhcA activity is controlled by SpiA via protein-protein interaction and iii) WhcA-SpiA interaction is controlled by the redox status of the cell (5). This mechanism is presumably conserved in *Streptomyces* (1). WhcE (WhiB1) is not essential. A *whcE* deletion mutant showed a prolonged lag-phase and an earlier cell death in the stationary growth phase (6). The σ^H sigma factor is encoded next to *whcE* and required for *whcE* transcription (7). WhcE plays an important role for survival under oxidative and heat stress (Table S5). Furthermore, it controls the expression of the thioredoxin reductase (*trxB*) (6) (Table S5). WhcB (WhiB3) is also not essential and seems to work together with WhcE, because overexpression of *whcB* complemented the Δ*whcE* phenotype (8). It seems to play a role during stationary phase growth, particularly in electron transfer reactions. (8). Potential target genes are phosphoglucomutase (cg2800), cysteine synthase (cg2833), NADH

oxidase (cg0404) and others (Table S5). WhcA, WhcB and WhcE influence each other in terms of expression and response to oxidative stress (9).

Deletion of WhcD (WhiB2) severely affects cell growth and leads to large filamentous, branched and bud-shaped morphology with multiple septa (10). Furthermore, *whcD* deletion affects fatty acid and mycolic acid synthesis (10) (Table S5). WhiD presumably regulates the expression of its target genes such as *ftsZ* by direct interaction with WhiA and thereby enhancing its DNA binding capability (11). This mechanism seems to be conserved in Streptomyces and Mycobacteria (1). In summary, the wbl proteins control various cellular functions, which are affected if the availability of [4Fe-4S] clusters is altered.

Supplemental Tables

Table S1: Oligonucleotides used in this study

Oligonucleotide	Sequence (5’ → 3’) and properties[a]
**Construction of deletion plasmid pK19mobsacB-Δ*trpP* and PCR-analysis of the resulting mutants**	
B453_TRP-D1	**AAAACGACGGCCAGTGAATT**AATGCCGAGCCATTTGCCAG
B454_TRP-D2	AACAACAACTCTATCCCCACCAATATTCC
Z225_TrpP-D3_new	**GTGGGGATAGAGTTGTTGTT**TAATTGAGACAAGCTTCCCAC
Z226_TrpP-D4_new	**CAGGTCGACTCTAGAGGA**GCTGCGTTACCACCGTGTTG
B457_TRP-Dfw	TAGAGCGCTTGGGATGCTCC
Z90_TrpP_Drv	GGGCACCTACCGAGGAAATC
**Construction of deletion plasmid pK19mobsacB-Δ*trpPv2* (TrpL$_{fbr}$ TrpE$_{S38R}$) and PCR-analysis of the resulting mutants[b]**	
B453_TRP-D1	**AAAACGACGGCCAGTGAATT**AATGCCGAGCCATTTGCCAG
B454_TRP-D2	AACAACAACTCTATCCCCACCAATATTCC
Z225_TrpP-D3_new	**GTGGGGATAGAGTTGTTGTT**TAATTGAGACAAGCTTCCCAC
Z226_TrpP-D4_new	**CAGGTCGACTCTAGAGGA**GCTGCGTTACCACCGTGTTG
B457_TRP-Dfw	TAGAGCGCTTGGGATGCTCC
Z90_TrpP_Drv	GGGCACCTACCGAGGAAATC
**Construction of deletion plasmid pK19*mobsacB*-ΔTRPv2_(*trpP)* and PCR-analysis of the resulting mutants**	
Z104_TRP-D1_neu	**AAAACGACGGCCAGTGAATT**GATCGGAATTATTCTTGGCAG
Z105_TRP-D2_neu	TTAATCATTTTTGGGTTCTTGC
Z106_TRP-D3_neu	**CAAGAACCCAAAAATGATTAA**GCCTTTAAATGTGGCAATGTTTCACG
B456_TRP-D4	**CAGGTCGACTCTAGAGGA**AGTATTTGGCGCCTTTGCCAAC
Z108_TRP-Dfw_neu	CGTGCAGTGAAGAATTCAGG
B458_TRP-Drv	TGGTCCCCCACTTCTTCACTGG
**Construction of deletion plasmid pK19mobsacB-Δ*aroP* and PCR-analysis of the resulting mutants**	
Z136_aroP_D1	**CAGGTCGACTCTAGAGGA**GGTGCCATCTGCCAGGTACG
Z137_aroP_D2	AGGCCGATAGAAATTATTCTGGAC
Z138_aroP_D3	**AGAATAATTTCTATCGGCCT**GTATCAACCGTAAACCCACA
Z139_aroP_D4	**AAAACGACGGCCAGTGAATT**CCGCATGGTCGACTATGTGG
Z140_aroP_dfw	GGTGACGCCAGCGGAAATGC
Z141_aroP_drv	CGTGGTGTGGACAAACAAGG
**Construction of deletion plasmid pK19mobsacB-Δ*sufR* and PCR-analysis of the resulting mutants**	
Z94_sufR-D1	**CAGGTCGACTCTAGAGGA**GTCGCGTACGACATCCTCGC
Z95_sufR-D2	TAAGCAGTAAGGCAATTTGC
Z96_sufR-D3	**GCAAATTGCCTTACTGCTTA**TGGTGTCACCTCCTGCTTG
Z97_sufR-D4	**AAAACGACGGCCAGTGAATT**GAAAAGCGTTGCCCCTAAAG
Z98_sufR-Dfw	TGAGGCGCTGCTGAAGCATC

Oligonucleotide	Sequence (5' → 3') and properties[a]
Z99_sufR-Drv	GCCATGCAAATCGGCGAATC
**Construction of deletion plasmid pK19mobsacB-SufR$_{L25P}$ & pK19mobsacB-SufR$_{Q193}$ and PCR-analysis of the resulting mutants[c]**	
Z100_sufR_SNP_D1	**CAGGTCGACTCTAGAGGA**TTGCCGAAGTCATGATCCGC
Z101_sufR_SNP_D2	ACCCATACGAGCCCAAAATG
Z102_sufR_SNP_D3	CATTTTGGGCTCGTATGGGTTCTGGCGCGGATTTGCCG
Z103_sufR_SNP_D4	**AAAACGACGGCCAGTGAATT**CCTCGCATCAGAGTCTGGTG
Z98_sufR-Dfw	TGAGGCGCTGCTGAAGCATC
Z99_sufR-Drv	GCCATGCAAATCGGCGAATC
**Construction of deletion plasmid pK19*mobsacB*-Δ*sufR*ΔP$_{sufB}$::P$_{tuf}$ and PCR-analysis of the resulting mutants**	
Z271_PsufB-D1	**AAAACGACGGCCAGTGAATT**GAAAAGCGTTGCCCCTAAAG
Z272_PsufB-D2	TGGTGTCACCTCCTGCTTG
Z273_PsufB-D3	ATGACTTCGGCAACGACGAACC
Z274_PsufB-D4	**CAGGTCGACTCTAGAGGA**AGATCTCAGGGTGCTCTTTC
Z275_PsufB-Ptuf-D5	**CAAGCAGGAGGTGACACCA**CACAGGGTAGCTGGTAGTTTG
Z276_PsufB-Ptuf-D6	**GTTCGTCGTTGCCGAA***GTCAT*TGTATGTCCTCCTGGACTTC
Z283_PsufB-Ptuf_Dfw	CAACGCCGTTGCCCTTAGGATTC
Z123_sufB_rv	ACTGCGAGTTCCGAGCTGAC
**Construction of deletion plasmid pK19*mobsacB*-Δ*sufR*ΔP$_{sufB}$::P$_{dapA}$ and PCR-analysis of the resulting mutants**	
Z271_PsufB-D1	**AAAACGACGGCCAGTGAATT**GAAAAGCGTTGCCCCTAAAG
Z272_PsufB-D2	TGGTGTCACCTCCTGCTTG
Z273_PsufB-D3	ATGACTTCGGCAACGACGAACC
Z274_PsufB-D4	**CAGGTCGACTCTAGAGGA**AGATCTCAGGGTGCTCTTTC
Z277_PsufB-PdapA-D5	**CAAGCAGGAGGTGACACCA**CGCAAAGCTCACACCCACGAG
Z278_PsufB-PdapA-D6	**GTTCGTCGTTGCCGAAGTCAT**AGAGTTCAAGGTTACCTTCT
Z284_PsufB-PdapA_Dfw	CATATAGTTAAGACAACATTTTTGGCTG
Z123_sufB_rv	ACTGCGAGTTCCGAGCTGAC
**Construction of expression plasmid pPREx2-*trpP* and PCR-analysis of the resulting plasmid**	
Z257_pPREX2-TrpP_D2	**GTAAAACGACGGCCAGTGAATT**TTAATCATTTTTGGGTTCTTGCGTAG
Z258_pPREX2-TrpP_D1	**GCAGAAGGAGATATACAT**ATGACGGTGATCGGAATTATTC
Z287_PRP113_pPREx2	CTTCTGGCGTCAGGCAGCCATC
Z288_PRP144	AGACCGCTTCTGCGTTCTG
**Construction of mutation plasmid pK19mobsacB-TrpD$_{A162E}$ and PCR-analysis of the resulting mutants with AvaI restriction site**	
Z159_TrpD fbr-D1	**AAAACGACGGCCAGTGAATT**CTCCAGCAACACTGAAAGTTC
Z160_TrpD fbr-D2	GCGCAATCTCGGGGTTGTACGCA
Z161_TrpD fbr-D3	**ACCCCGAGATTGCGC**ATGTGCAGCCGG
Z162_TrpD fbr-D4	**CAGGTCGACTCTAGAGGA**GGTGCCGTCGGCAAGCAAGG
Z163_TrpD fbr-fw	AACAGCTTCTCGCGAACTAAT
Z164_TrpD fbr-rv	TCGACGATGCTTTCCAACAC

Oligonucleotide	Sequence (5' → 3') and properties[a]
**Construction of plasmid pJC1-P_{sufR}-venus and PCR-analysis of the resulting strains**	
Z192_sufR-eYFP-regular_P1	**GATCAGCGACGCCGCAGGGGGCTGATCTGGAACCAGCGGT**
Z193_sufR-eYFP-regular_P2	TGGTGTCACCTCCTGCTTGATTTAGCTTTCACCTTTAGACAACACAAGTGTTCCCTAATTC
Z194_sufR-eYFP-regular_P3	GAATTAGGGAACACTTGTGTTGTCTAAAGGTGAAAGCTAAATCAAGCAGGAGGTGACACCA**ATGGTGAGCAAGGGCGAGGAGC**
Z195_sufR-eYFP-regular_P4	GTTGCCATTGCTGCAGGTCGA**TTATCTAGACTTGTACAGCTC**
Z200_pJC1_MCS-fw	CAGGGACAAGCCACCCGCACA
Z201_pJC1_MCS-rv	GGAAGCTAGAGTAAGTAGTTCGC
**Construction of plasmid pJC1-P_{sufR}-venus-Mut_{TSS1} and PCR-analysis of the resulting strains**	
Z192_sufR-eYFP-regular_P1	**GATCAGCGACGCCGCAGGGGGCTGATCTGGAACCAGCGGT**
Z196_sufR-eYFP-mut1_P2	TGGTGTCACCTCCTGCTTGATTTAGCTTTCACCTTTAGACAAGTAAAGTGTTCCCTAATTC
Z197_sufR-eYFP-mut1_P3	GAATTAGGGAACACTTTACTTGTCTAAAGGTGAAAGCTAAATCAAGCAGGAGGTGACACCA**ATGGTGAGCAAGGGCGAGGAGC**
Z195_sufR-eYFP-regular_P4	GTTGCCATTGCTGCAGGTCGA**TTATCTAGACTTGTACAGCTC**
Z200_pJC1_MCS-fw	CAGGGACAAGCCACCCGCACA
Z201_pJC1_MCS-rv	GGAAGCTAGAGTAAGTAGTTCGC
**Construction of plasmid pJC1-P_{sufR}-venus- Mut_{TSS2} and PCR-analysis of the resulting strains**	
Z192_sufR-eYFP-regular_P1	**GATCAGCGACGCCGCAGGGGGCTGATCTGGAACCAGCGGT**
Z198_sufR-eYFP-mut2_P2	TGGTGTCACCTCCTGCTTGATTTAGCTTTCACCTTTAGACAACACAAGTCAGCCCTAATTC
Z199_sufR-eYFP-mut2_P3	GAATTAGGGCTGACTTGTGTTGTCTAAAGGTGAAAGCTAAATCAAGCAGGAGGTGACACCA**ATGGTGAGCAAGGGCGAGGAGC**
Z195_sufR-eYFP-regular_P4	GTTGCCATTGCTGCAGGTCGA**TTATCTAGACTTGTACAGCTC**
Z200_pJC1_MCS-fw	CAGGGACAAGCCACCCGCACA
Z201_pJC1_MCS-rv	GGAAGCTAGAGTAAGTAGTTCGC

[a] Overlaps for Gibson assembly are written in bold letters. Restriction sites are underlined.

[b] Template: DNA isolated from the strain WT TRP^{+}.

[c] Template: gDNA from the isolated mutant strains. Construct with mutated NdeI restriction site.

Table S2: Topology of TrpP

Tool	N-terminus	TM1	TM2	TM3	C-Terminus
Topcons[a] (12)	OUT	6-26	58-78	84-104	IN
Phobius (13)	OUT	6-29	61-85	91-112	IN
DeepTMHMM (14)	OUT	7-27	59-79	82-106	IN
TMHMM 2.0 (15)	IN	7-29	57-79	84-106	OUT

[a] Includes five different prediction tools: Octopus, Philius, Polyphobius, Scampi, and Spoctopus

Table S3: Transcriptomic comparison of WT Δ*trpP* and WT [a]

Locus-tag	Gene name	Annotation	Ratio	n	p-value	Regulated by[b]
cg0229	*gltB*	glutamine 2-oxoglutarate aminotransferase NADPH large subunit, also glutamate synthase (EC:1.4.1.13)	4.765	4	0.017	AmtR, GlxR(P), ArgR(P)
cg1216	*nadA*	quinolinate synthetase	3.991	4	0.002	NdnR, WhcA(P)
cg1628		putative hydrolase of the α/β superfamily	3.989	4	0.004	
cg1218	*ndnR*	transcriptional repressor of NAD de novo biosynthesis genes *ndnR-nadA-nadC-nadS* operon, NrtR-family	3.790	4	0.010	WhcA(P), NdnR
cg1626		hypothetical protein, conserved	3.709	4	0.026	
cg1785	*amtA*	high-affinity ammonia permease	3.521	4	0.005	AmtR
cg0230	*gltD*	glutamine 2-oxoglutarate aminotransferase NADPH small subunit, also glutamate synthase (EC:1.4.1.13)	3.090	4	0.001	AmtR, GlxR(P), ArgR(P)
cg1214	*nadS*	cysteine desulfurase-like protein involved in Fe-S cluster assembly, required for maturation of NadA	2.969	4	0.001	WhcA(P), NdnR
cg1784	*ocd*	ornithine cyclodeaminase (EC:4.3.1.12)	2.909	4	0.005	AmtR
cg1299	*cydD*	ABC transporter, subunit II, essential for cytochrome *bd* oxidase assembly	2.857	4	0.005	OxyR
cg1301	*cydA*	cytochrome *bd* oxidase, subunit I	2.769	4	0.000	OxyR
cg1215	*nadC*	quinolinate phosphoribosyltransferase	2.703	4	0.001	WhcA(P), NdnR
cg1300	*cydB*	cytochrome *bd* oxidase, subunit II	2.603	4	0.002	OxyR
cg2047	-	putative secreted protein CGP3 region	2.198	4	0.014	
cg1298	*cydC*	ABC transporter, subunit I, essential for cytochrome *bd* oxidase assembly	2.192	4	0.008	OxyR
cg2261	*amtB*	low affinity ammonium uptake protein	2.159	4	0.022	GlxR(P), AmtR
cg0905	*psp2*	putative secreted protein	2.077	4	0.003	
cg2407			2.077	4	0.004	
cg1139	-	putative allophanate hydrolase subunit 2	0.500	4	0.013	GlxR(P), Cg1143(P)
cg1920	-	hypothetical protein CGP3 region	0.495	4	0.012	
cg1230	-	hypothetical protein, conserved	0.493	4	0.023	
cg1921	-	hypothetical protein CGP3 region	0.489	4	0.009	
cg1923	-	hypothetical protein CGP3 region	0.488	4	0.036	
cg1140	-	putative allophanate hydrolase subunit 1	0.488	4	0.018	GlxR(P), Cg1143(P)
cg1595	*uspA2*	universal stress protein no. 2, nucleotide-binding protein	0.484	4	0.006	GlxR(P)
cg0922	-	putative secreted siderophore-binding lipoprotein	0.481	4	0.012	DtxR
cg1412	*rbsC*	ribose/xylose transporter, ABC-type transport system, permease component (TC 3.A.1.2.1)	0.478	4	0.010	UriR, RbsR
cg1310	*rolM (tfdF)*	maleylacetate reductase (EC:1.3.1.32)	0.476	4	0.012	RolR, GlxR(P)
cg0097	-	putative zinc finger protein, conserved	0.476	4	0.008	BioQ
cg1922	-	hypothetical protein CGP3 region	0.475	4	0.015	
cg1919	-	putative membrane protein CGP3 region	0.474	4	0.009	
cg0202	*iolD*	3,5/4-trihydroxycyclohexa-1,2-dione hydrolase, TPP-requiring (EC 4.1.3.18)	0.468	4	0.016	GlxR, IolR
cg3395	*proP*	proline/ectoine carrier, MFS-type	0.467	4	0.005	MtrA, OxyR
cg1924	-	hypothetical protein CGP3 region	0.460	4	0.014	
cg1231	*chaA*	$Na^+(K^+)/H^+$ antiporter	0.455	4	0.023	
cg0201	*iolB*	enzyme involved in inositol metabolism	0.449	4	0.013	GlxR, IolR
cg3385	*catA3 (rhcD2)*	catechol 1,2-dioxygenase (EC:1.13.11.37)	0.446	4	0.011	Cg3388(P)
cg1226	*pobB (pobA)*	4-hydroxybenzoate 3-monooxygenase (EC:1.14.13.2)	0.441	4	0.003	GlxR(P), PcaO(P), PcaR(P)

Locus-tag	Gene name	Annotation	Ratio	n	p-value	Regulated by[b]
cg1225	*benK3 (pcaK)*	putative benzoate transport transmembrane protein	0.440	4	0.007	GlxR(P), PcaO(P), PcaR(P)
cg1142	-	putative Na^+/proline, Na^+/panthothenate symporter	0.440	4	0.013	GlxR(P), Cg1143(P)
cg3047	*ackA*	acetate kinase (EC:2.7.2.1)	0.435	4	0.011	RamA, RipA, RamB, GlxR(P)
cg0921	-	putative cytoplasmic siderophore-interacting protein	0.433	4	0.016	DtxR
cg0205	*iolH*	*myo*-inositol catabolism protein	0.426	4	0.003	GlxR, IolR
cg0254	-	putative amino acid carrier protein sodium/alanine symporter	0.424	4	0.012	
cg0347	*phdE (hdtZ)*	enoyl-CoA hydratase; involved in degradation of aromatic compounds	0.423	4	0.002	PhdR, GlxR(P)
cg3374	*cye1*	putative NADH-dependent flavin oxidoreductase, Old Yellow Enzyme family, probably involved in oxidative stress response	0.423	4	0.000	McbR(P), CyeR, CysR(P)
cg2937	*siaE*	ABC-Transporter for sialic acid, secreted component	0.419	4	0.010	NanR
cg0204	*iolG*	putative oxidoreductase, *myo*-inositol 2-dehydrogenase (EC:1.1.1.18)	0.416	4	0.002	GlxR, IolR
cg2940	*siaI*	ABC-transporter for sialic acid, contain duplicated ATPase domains	0.415	4	0.003	NanR
cg1109	*porB*	anion-specific porin precursor	0.414	4	0.003	PhoR
cg2181	*oppA*	ABC-type peptide transport system, secreted component	0.408	4	0.013	AmtR
cg0344	*phdB (fabG1)*	3-hydroxyacyl-CoA dehydrogenase; involved in degradation of aromatic compounds	0.408	4	0.006	PhdR, GlxR(P)
cg3386	*tcbF (rhcM2)*	maleylacetate reductase (EC:1.3.1.32)	0.408	4	0.009	Cg3388(P)
cg0134	*abgB*	metal-dependent amidase/aminoacylase/carboxypeptidase, AbgB homolog	0.406	4	0.004	MtrA
cg0203	*iolE*	2-keto-*myo*-inositol dehydratase (EC:4.2.1.44)	0.405	4	0.003	GlxR, IolR
cg3127	*tctC*	citrate uptake transporter, substrate binding protein	0.403	4	0.012	GlxR(P), CitB
cg0345	*phdC*	3-oxoacyl-CoA ketohydrolase (acetyl-CoA forming); involved in degradation of aromatic compounds	0.399	4	0.011	PhdR, GlxR(P)
cg2628	*pcaC*	γ-carboxymuconolactone decarboxylase (EC:4.1.1.44)	0.388	4	0.004	PcaR(P), GlxR(P), PcaO
cg0346	*phdD (fadE)*	acyl-CoA dehydrogenase; involved in degradation of aromatic compounds	0.384	4	0.003	PhdR, GlxR(P)
cg2629	*pcaB*	β-carboxy-cis,cis-muconate cycloisomerase (EC:5.5.1.2)	0.381	4	0.003	GlxR(P), PcaO, PcaR(P)
cg2939	*siaG*	ABC-transporter for sialic acid, fused permease and ATPase components	0.372	4	0.012	NanR
cg3387	*iolT2*	*myo*-Inositol transporter 2, MFS-type	0.368	4	0.007	Cg3388(P)
cg0212	-	putative phosphate isomerase/epimerase, conserved	0.358	4	0.008	
cg0637	*creC*	putative NAD^+-dependent 4-hydroxybenzaldehyd dehydrogenase subunit (EC: 1.2.1.64)	0.358	4	0.002	GenR(P), SugR(P)
cg0198	-	putative protein, conserved, probably involved in *myo*-inositol metabolism	0.355	4	0.009	GlxR, IolR
cg0199	*iolA*	aldehyde dehydrogenase, methylmalonate-semialdehyde dehydrogenase (EC:1.2.1.27), *myo*-inositol catabolism	0.354	4	0.009	GlxR, IolR
cg2938	*siaF*	ABC-Transporter for sialic acid, permease component	0.350	4	0.015	NanR
cg0638	*creD*	p-cresol methylhydroxylase subunit	0.344	4	0.001	GenR(P), SugR(P)
cg2560	*aceA*	isocitrate lyase (EC:4.1.3.1), part of glyoxylate shunt	0.343	4	0.000	RamA, RamB, GlxR
cg2559	*aceB*	malate synthase (EC:2.3.3.9), part of glyoxylate shunt	0.336	4	0.003	GlxR, RamA, CspA2, RamB
cg0211	*oxiB*	putative oxidoreductase dehydrogenase	0.334	4	0.005	

Locus-tag	Gene name	Annotation	Ratio	n	p-value	Regulated by[b]
cg0639	*creE*	ferredoxin reductase, part of putative cytochrome P450 system of p-cresol methylhydroxylase	0.332	4	0.002	GenR(P), SugR(P)
cg0133	*abgT*	p-aminobenzoyl-glutamate transporter	0.324	4	0.005	MtrA
cg1090	*ggtB*	γ-glutamyltranspeptidase precursor PR (EC:2.3.2.2)	0.322	4	0.001	
cg0645	*creJ (cytP)*	cytochrome P450 (EC:1.14.15.1), part of putative cytochrome P450 system of p-cresol methylhydroxylase	0.322	4	0.001	GenR(P), SugR(P)
cg2630	*pcaG*	protocatechuate dioxygenase α subunit (EC:1.13.11.3)	0.317	4	0.005	GlxR(P), PcaO, PcaR(P)
cg3126	*tctB*	citrate uptake transporter, membrane subunit	0.317	4	0.015	GlxR(P), CitB
cg2636	*catA1 (catA)*	catechol 1,2-dioxygenase (EC:1.13.11.1)	0.310	4	0.002	RipA, GlxR(P), BenR(P)
cg3125	*tctA*	citrate uptake transporter, membrane subunit	0.309	4	0.012	CitB, GlxR(P)
cg0088	*citH*	citrate transporter, CitMHS-family	0.308	4	0.001	GlxR, CitB
cg2631	*pcaH*	protocatechuate dioxygenase β subunit (EC:1.13.11.3)	0.307	4	0.002	PcaR(P), GlxR(P), PcaO
cg2837	*sucC*	succinyl-CoA synthetase subunit β, ADP-forming (EC:6.2.1.5)	0.288	4	0.009	RamA, RamB(P), GlxR
cg0640	*creF (fdxB)*	ferredoxin no. 2, 2Fe-2S, part of putative cytochrome P450 system of p-cresol methylhydroxylase	0.281	4	0.004	GenR(P)
cg2966	-	putative phenol 2-monooxygenase (EC:1.14.13.7)	0.274	4	0.002	cg2965(P), GlxR(P)
cg0642	*creH*	putative PEP-utilizing enzyme, probably DNA binding, conserved	0.270	4	0.002	GenR(P), SugR(P)
cg0641	*creG (fabG2)*	4-hydroxybenzyl-alcohol dehydrogenase (EC: 1.17.99.1)	0.246	4	0.001	GenR(P), SugR(P)
cg3216	*gntP*	gluconate permease, gluconate:H^+ symporter GntP-family	0.236	4	0.002	GlxR, GntR1, GntR2
cg0223	*iolT1*	*myo*-inositol transporter 1	0.233	4	0.002	IolR
cg0644	*creI*	putative pyruvate phosphate dikinase, PEP/pyruvate binding	0.220	4	0.005	SugR(P), GenR(P)
cg2836	*sucD*	succinyl-CoA synthetase α subunit, ADP-forming (EC:6.2.1.5)	0.214	4	0.000	RamB(P), GlxR(P), RamA
cg1309	*rolH*	3-3-hydroxyphenylpropionate hydroxylase or 2-polyprenyl-6-methoxyphenol hydroxylase and related FAD-dependent oxidoreductase	0.213	4	0.002	RolR, GlxR(P)
cg0961	-	putative homoserine O-acetyltransferase	0.213	4	0.001	GlxR(P)
cg2312	*gip*	putative hydroxypyruvate isomerase (EC:5.3.1.22), maybe involved in glyoxylate and dicarboxylate metabolism	0.197	3	0.006	cg2314(P)
cg2616	*vanA*	vanillate demethylase, oxygenase subunit	0.160	4	0.002	VanR, GlxR(P)
cg1612	-	putative acetyltransferase	0.159	4	0.002	MtrA
cg2610	-	putative ABC-type dipeptide/oligopeptide/nickel transport system, secreted component	0.134	4	0.001	GlxR
cg3096	*ald*	aldehyde degyhdrogenase, essential for ethanol as sole carbon source	0.133	4	0.001	RamA, RamB, GlxR(P)
cg3195	-	putative flavin-containing monooxygenase FMO	0.089	4	0.000	GlxR, IpsA
cg3107	*adhA*	Zn-dependent alcohol dehydrogenase (EC:1.1.1.1)	0.084	4	0.000	AtlR, RamB, RamA, GlxR(P)
cg3357	*trpP*	tryptophan-specific permease, 5-methyltryptophan resistance	0.068	4	0.000	

[a] Results of microarray experiments comparing the gene expression in WT Δ*trpP* vs. WT. Shown are all target genes with at least 2-fold change in at least 3 of 4 experiments (p-value < 0.05) and a signal to noise ratio of >2.
[b] (P) indicates that a regulatory interaction is predicted. All other regulatory interactions given are experimentally proven.

Table S4: Mutations identified in evolved strains

Strain	Position & mutation on DNA level	Locus tag	Mutation
C1* ΔTRP Δ*trpP* evo1	SNV A1481472G	cg1765	SufR_{L25P}
C1* ΔTRP Δ*trpP* evo2	SNV T1481317C	cg1765	SufR_{R77G}
C1* ΔTRP Δ*trpP* evo3	SNV G1480969A	cg1765	SufR_{Q193*}

Genome sequencing was performed with three independent clones isolated from the 16th batch of the ALE experiment. Reads were mapped using Genbank accession CP017995 as the reference genome. Single nucleotide variants (SNV) are given for the plus strand.

Table S5: WhiB-like proteins in *C. glutamicum* and their potential target genes.

Locus Tags	Gene name	Annotation	Potential target genes	Ref.
cg0337 NCgl0275	*whcA* (w*hiB4*)	WhiB homolog, plays a negative role in SigH-mediated oxidative stress response. Protein-protein interaction with SpiA (cg1068, NCgl0899).	cg0404 (*noxA*), cg1214 (*nadS*), cg1215 (*nadC*), cg1216 (*nadA*), cg1218 (*ndnR*), cg2342, cg3405 cg3424 (*cwlM*), cg3422 (*trxB*), cg3423 (*trxC*)	(3-5)
cg0695 NCgl0574	*whcB* (w*hiB3*)	WhiB homolog, regulator involved in oxidative stress response, negative regulation of trxB.	cg0404 (*noxA*), cg1423, cg1451 (*serA*), cg1763 (*sufD*), cg2091 (*ppgK*), cg2800 (*pgm*), cg2833 (*cysK*), cg3114 (*cysN*), cg3237 *(sodA*), cg3422 (*trxB*)	(8)
cg0850 NCgl0711	*whcD* (*whiB2*)	trancriptional regulator, WhiB homolog, involved in the early stages of cell division and fatty acid synthesis. Requires WhiA (cg 1792, NCgl1527) for DNA binding.	cg0812 (*accD1*), cg0957 (*fas-IB*), cg2363 (*sepF*), cg2366 (*ftsZ*), cg2367 (*ftsQ*), cg2361 (*divIVA*), cg2375 (*ftsI*), cg2743 (*fas-IA*),	(10, 11)
cg0878 NCgl0734	*whcE* (*whiB1*)	WhiB homolg, transcriptional regulator, positive role in survival under heat and oxidative stress	cg3422(*trxB*)	(6)

Supplemental Figures

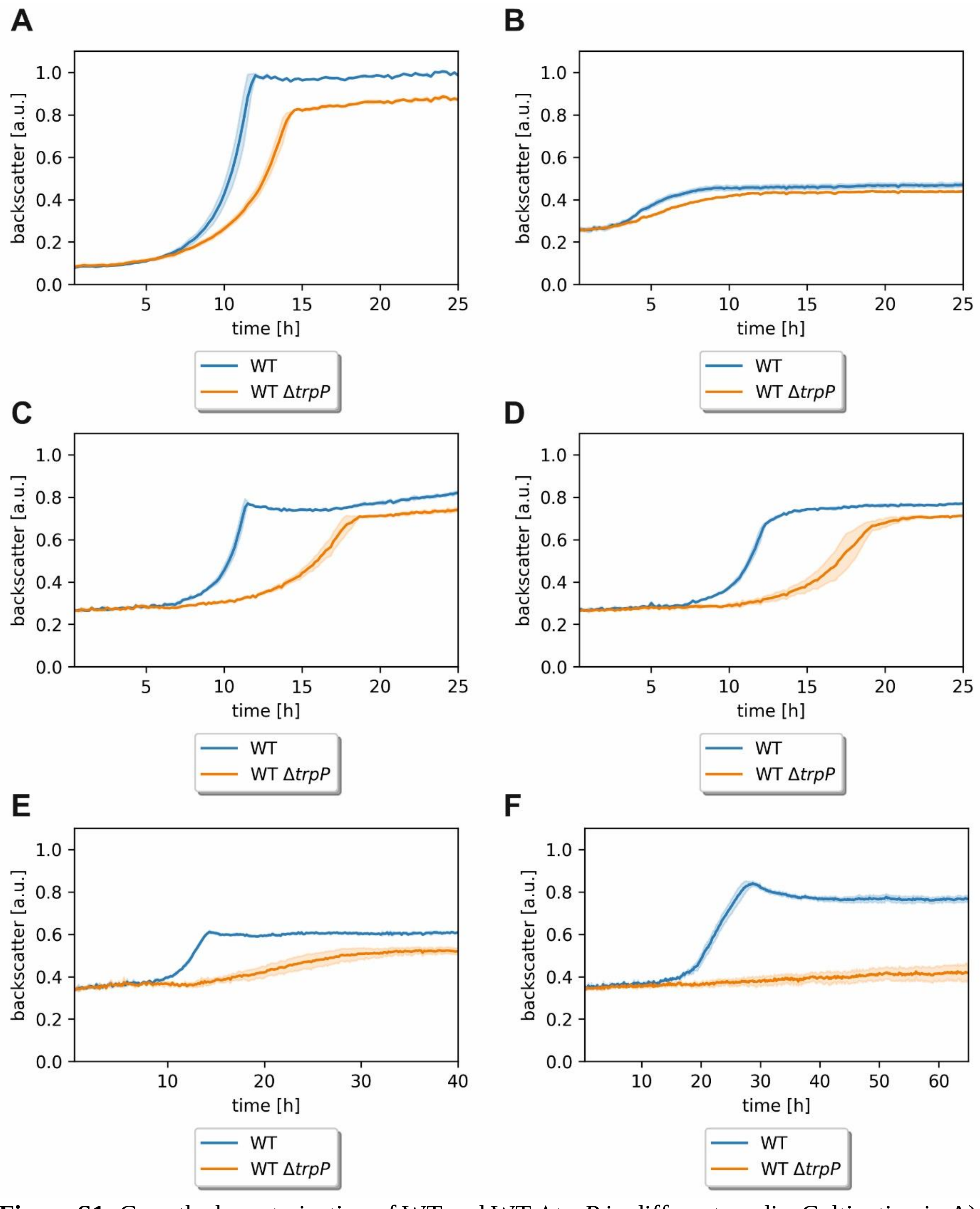

Figure S1: Growth characterization of WT and WT Δ*trpP* in different media. Cultivation in A) CGXII medium with 111 mM D-glucose, B) Brain-heart-infusion complex medium (Difco™), C) CGXII medium with 100 mM gluconate and D) CGXII medium with 100 mM *myo*-inositol, E) CGXII medium with 200 mM acetate, F) CGXII medium with 400 mM acetate. All cultures were performed in triplicate Based on the maximum value recorded for WT in CGXII with glucose, the backscatter data were normalized. The mean values are shown as lines and standard deviations as shaded areas.

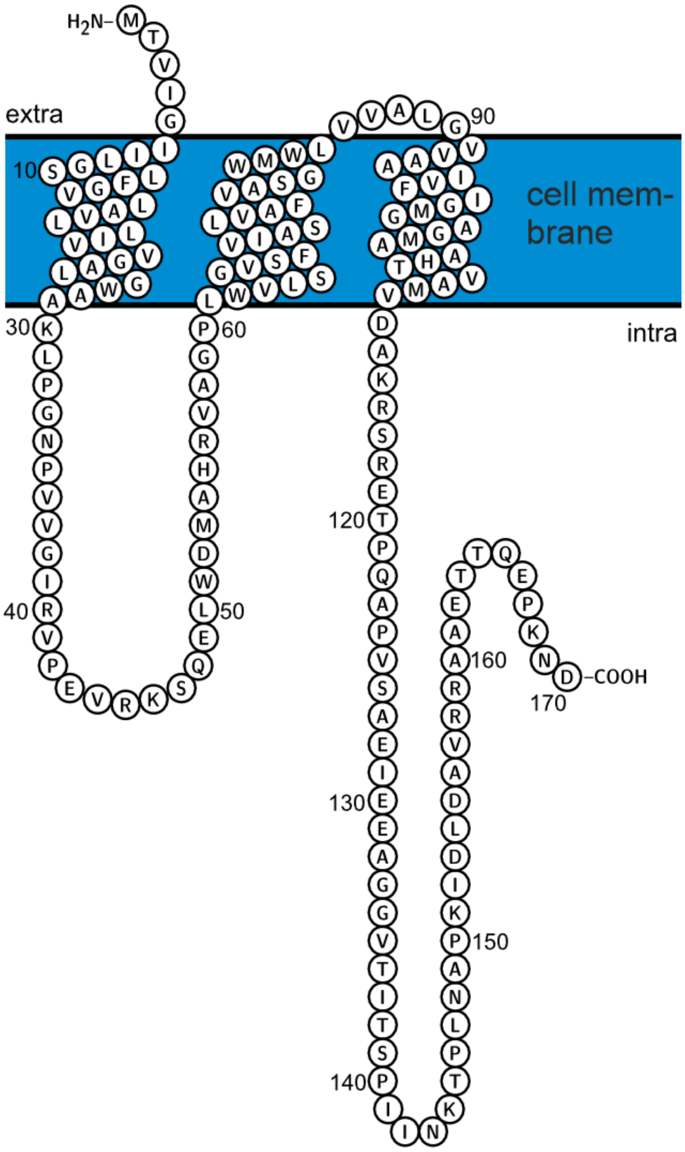

Figure S2: Topology model of TrpP, generated with Protter (16).

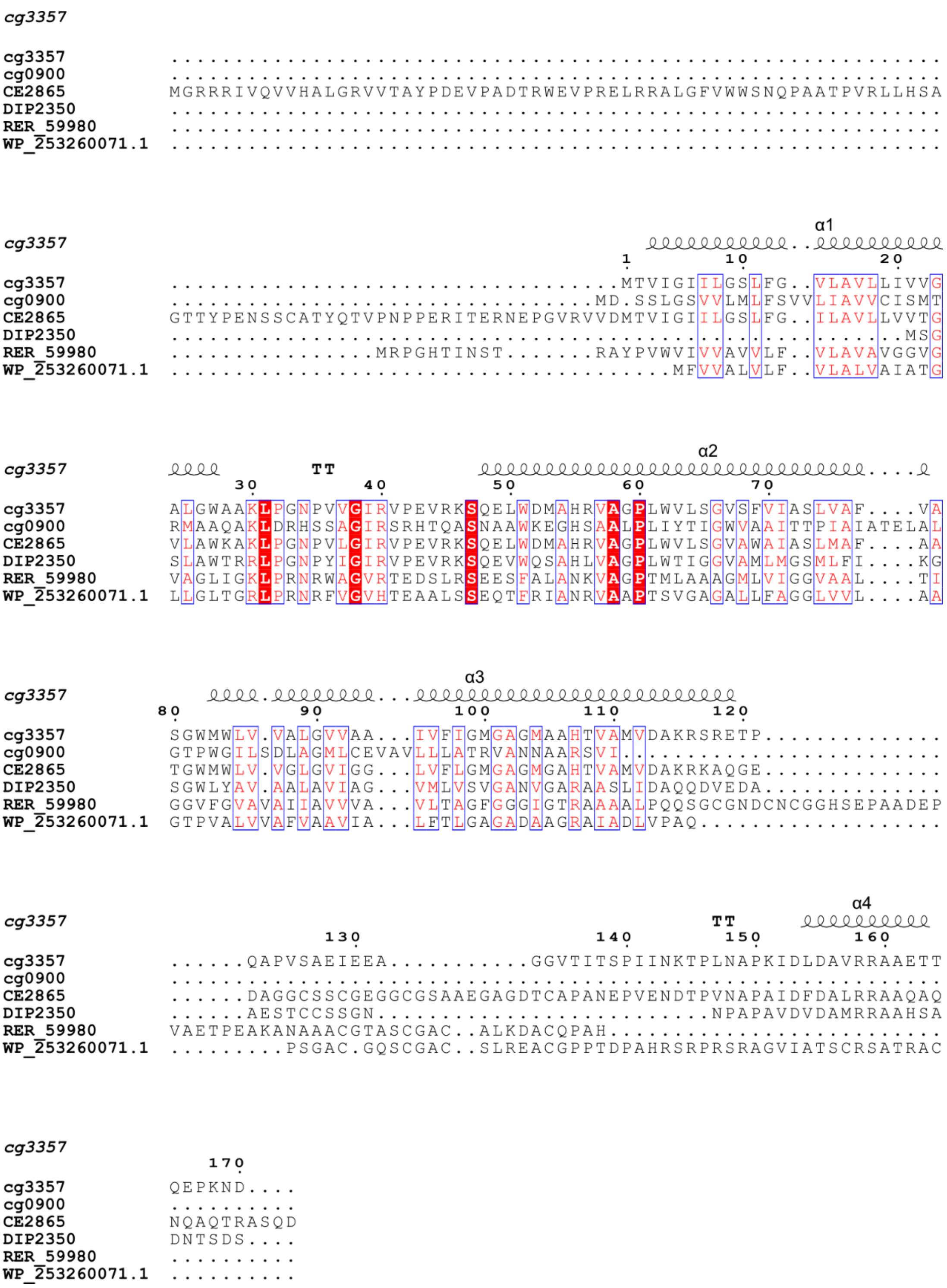

Figure S3: Multiple sequence alignment of TrpP (cg3357) of *C. glutamicum* (NCBI accession: CAF18968) with homologous sequences: cg0900 of *C. glutamicum* (CAF19495), CE2865 of *Corynebacterium efficiens* (BAC19675), DIP2350 of *Corynebacterium diphtheriae* (CAE50873), RER_59980 of *Rhodococcus erythropolis* (BAH36706) and SdpI-family protein of *Nocardia farcinica* (WP_253260071). Clustal Omega was used for the sequence alignment (17). The alignment was visualized with ESPribt 3.0 (18). Sequence numbering and structure prediction according to the AlphaFold model of cg3357 (19, 20). Residues with high similarity are marked as red colored letters, and identical residues via a red background color. Structural data are assigned (helices: squiggles, turns: T).

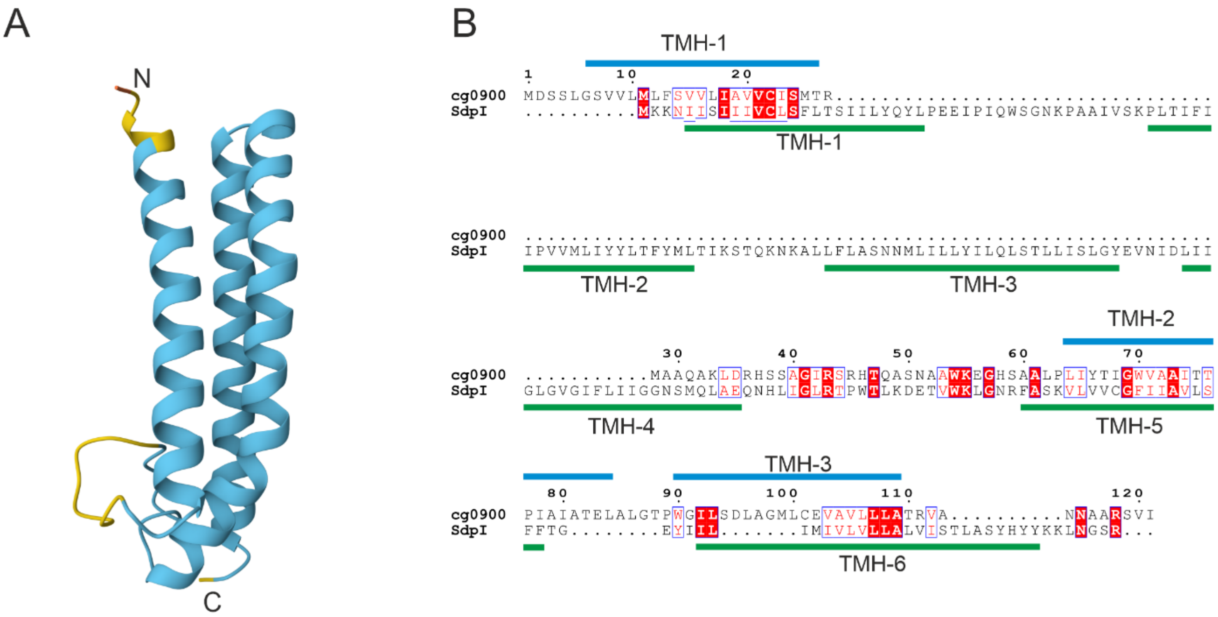

Figure S4: AlphaFold model of *C. glutamicum* Cg0900 and alignment with SdpI. A) AlphaFold model of Cg0900. N and C indicate the N- and C-terminus of the protein. The N-terminus is predicted to be extracellular, the C-terminus is predicted to be intracellular. The protein chain color represents the model confidence from dark blue (very high), along light blue (high) and yellow (low) to red (very low) (19, 20). B) Sequence alignment of *C. glutamicum* Cg0900 and *B. subtilis* SdpI, prepared with Clustal Omega (17) and visualized with ESPribt 3.0 (18). Transmembrane helices according to Phobius (13) are given for Cg0900 in blue and for SdpI in green. Residues with high similarity are marked as red colored letters, and identical residues via a red background color.

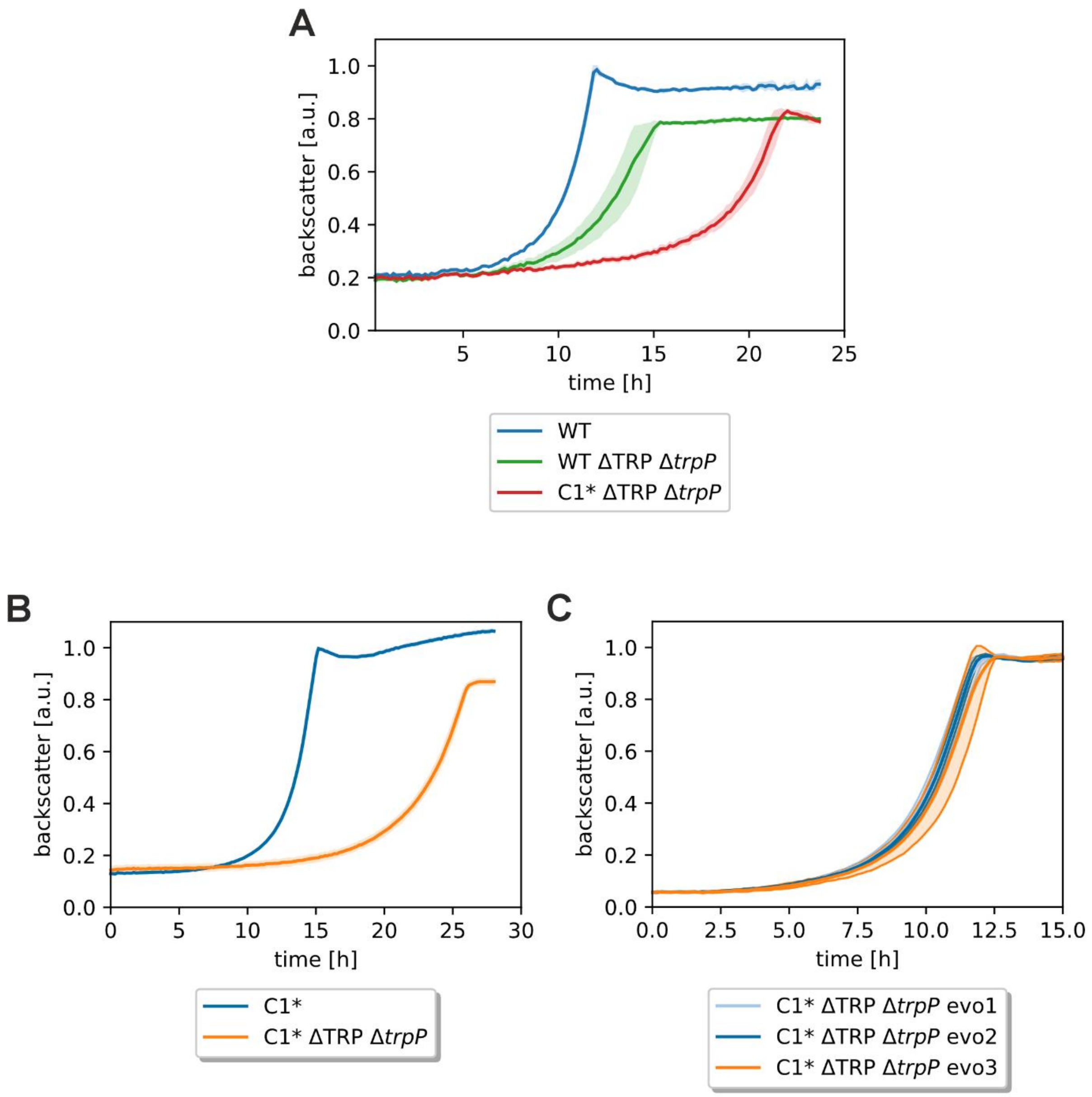

Figure S5: Growth of ALE strains before and after the evolution. A, Comparison of WT ΔTRP Δ*trpP* and the genome reduced strain C1*ΔTRP Δ*trpP* that was used for the ALE. B, comparison of the ALE start strain C1*ΔTRP Δ*trpP* with the parental strain C1*. C, Growth of the three evolved strains after the ALE. All cells were cultivated in the BioLector in CGXII minimal medium with 111 mM glucose at 30 °C, 1400 rpm. Auxotrophic strains were supplemented with 0.5 mM (A) or 0.5 g L^{-1} (B, C) of L-tryptophan. Backscatter data were normalized by the maximum recorded value in the run. A and B, average of three biological replicates, C, average of two biological replicates. The standard deviation is shown as shaded area.

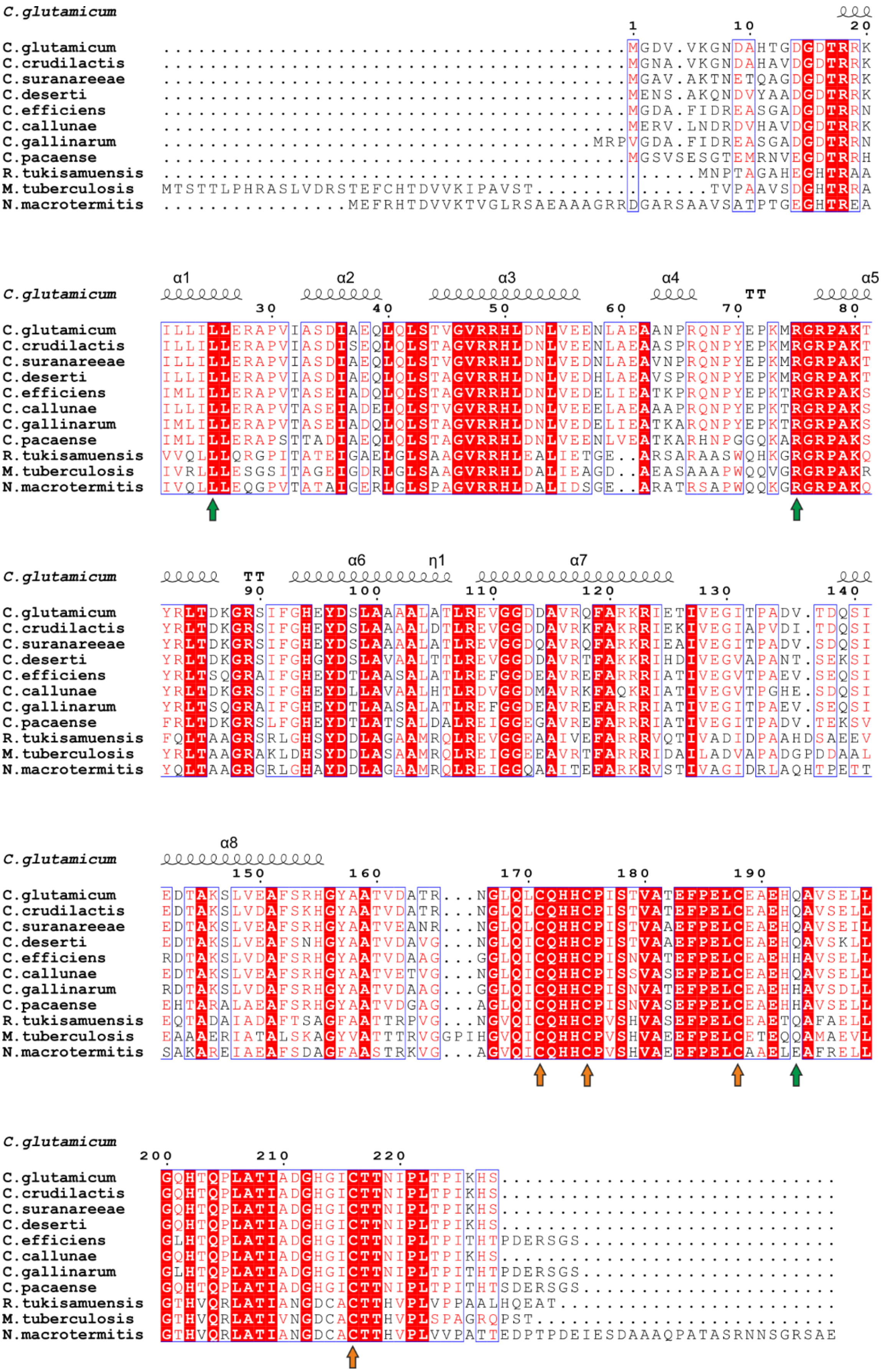

Figure S6: Multiple sequence alignment of SufR from *C. glutamicum* (WP_003862282.1) with homologous sequences of *C. pacaense* (WP_080794855.1), *C. efficiens* (WP_035108821.1),

C. gallinarum (WP_191733600.1), *C. callunae* (AGG66925.1), *C. deserti* (WP_053544972.1), *C. crudilactis* (WP_066569703.1), *C. suranareeae* (BAU95912.1), *Mycobacterium tuberculosis* (WP_096759740.1), *Rhodococcus tukisamuensis* (WP_245709563.1), and *Nocardia macrotermitis* (WP_153411708.1). Clustal Omega was used for the sequence alignment (17). Via Swissmodel, a potential structure of *C. glutamicum* SufR was predicted as reference (modeled after the crystal structure of RecX PDB 3d5l.1.A) (21). The alignment was visualized with ESPribt 3.0 (18). Residues with high similarity are marked as red colored letters, and identical residues via a red background color. Structural data are assigned (helices: squiggles, β-strands: arrows, turns: T). The mutation sites identified in the evolved *C. glutamicum* strains are marked with green arrows. The presumed 4Fe-4S cluster coordinating residues at positions 171, 175, 188, and 216 are marked with orange arrows.

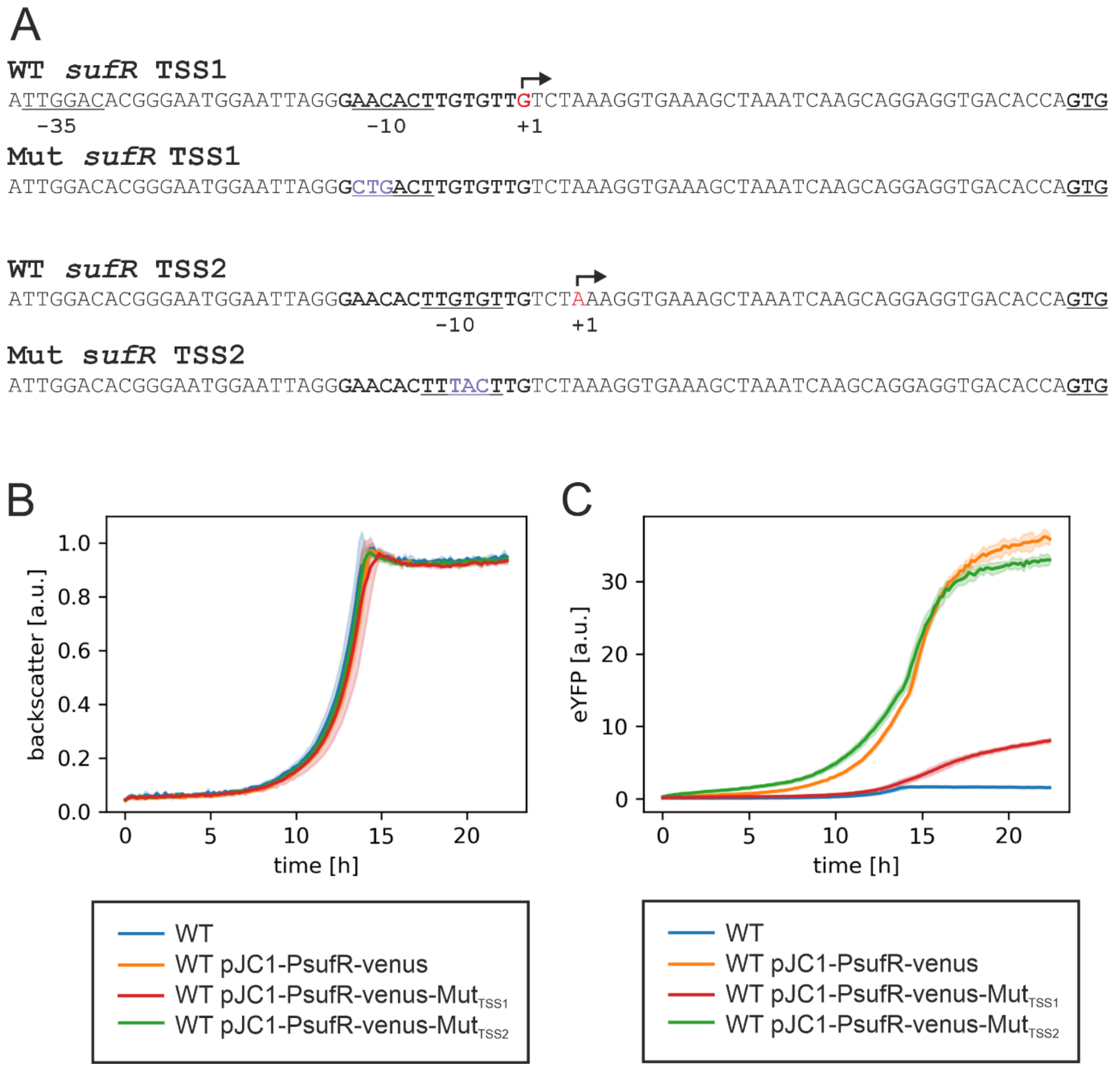

Figure S7: Analysis of the importance of two *sufR* transcriptional start sites (TSS). (A) Promoter region of *sufR* (cg1765) in *C. glutamicum* with two TSS marked in red and with arrow. The -10 and -35 regions are underlined. The underlined "GTG" is the SufR start codon. The predicted SufR binding site is shown in bold letters. The sequences with mutations are shown below the native sequence with the mutated bases in purple. (B) Growth of the WT with different pJC1 plasmids carrying either the native or a mutated version of the SufR promoter fused to the mVenus coding sequence. (C) Fluorescence of the same strains shown in (A). Cultivation in CGXII medium with 111 mM glucose at 1400 rpm, 85% humidity and 30°C in the BioLector. The backscatter data were normalized based on the maximum value recorded for each WT in the respective experiment. The mean values are shown as lines and standard deviations as shaded areas.

References

1. Bush MJ. The actinobacterial WhiB-like (Wbl) family of transcription factors. Mol Microbiol. 2018;110(5):663-76.
2. Sharma V, Hardy A, Luthe T, Frunzke J. Phylogenetic distribution of WhiB- and Lsr2-type regulators in actinobacteriophage genomes. Microbiol Spectr. 2021;9(3):e0072721.
3. Choi WW, Park SD, Lee SM, Kim HB, Kim Y, Lee HS. The *whcA* gene plays a negative role in oxidative stress response of *Corynebacterium glutamicum*. FEMS Microbiol Lett. 2009;290(1):32-8.
4. Park JS, Shin S, Kim ES, Kim P, Kim Y, Lee HS. Identification of SpiA that interacts with *Corynebacterium glutamicum* WhcA using a two-hybrid system. FEMS Microbiol Lett. 2011;322(1):8-14.
5. Park JS, Lee JY, Kim HJ, Kim ES, Kim P, Kim Y, et al. The role of *Corynebacterium glutamicum spiA* gene in *whcA*-mediated oxidative stress gene regulation. FEMS Microbiol Lett. 2012;331(1):63-9.
6. Kim TH, Park JS, Kim HJ, Kim Y, Kim P, Lee HS. The *whcE* gene of *Corynebacterium glutamicum* is important for survival following heat and oxidative stress. Biochem Biophys Res Commun. 2005;337(3):757-64.
7. Kim TH, Kim HJ, Park JS, Kim Y, Kim P, Lee HS. Functional analysis of *sigH* expression in *Corynebacterium glutamicum*. Biochem Biophys Res Commun. 2005;331(4):1542-7.
8. Lee JY, Park JS, Kim HJ, Kim Y, Lee HS. *Corynebacterium glutamicum whcB*, a stationary phase-specific regulatory gene. FEMS Microbiol Lett. 2012;327(2):103-9.
9. Lee JY, Kim HJ, Kim ES, Kim P, Kim Y, Lee HS. Regulatory interaction of the *Corynebacterium glutamicum whc* genes in oxidative stress responses. J Biotechnol. 2013;168(2):149-54.
10. Lee DS, Kim Y, Lee HS. The *whcD* gene of *Corynebacterium glutamicum* plays roles in cell division and envelope formation. Microbiology. 2017;163(2):131-43.
11. Lee DS, Kim P, Kim ES, Kim Y, Lee HS. *Corynebacterium glutamicum* WhcD interacts with WhiA to exert a regulatory effect on cell division genes. Antonie Van Leeuwenhoek. 2018;111(5):641-8.
12. Tsirigos KD, Peters C, Shu N, Käll L, Elofsson A. The TOPCONS web server for consensus prediction of membrane protein topology and signal peptides. Nucleic Acids Res. 2015;43(W1):W401-W7.
13. Kall L, Krogh A, Sonnhammer ELL. Advantages of combined transmembrane topology and signal peptide prediction-the Phobius web server. Nucleic Acids Res. 2007;35(Web Server):W429-W32.
14. Hallgren J, Tsirigos KD, Pedersen MD, Armenteros JJA, Marcatili P, Nielsen H, et al. DeepTMHMM predicts alpha and beta transmembrane proteins using deep neural networks. bioRxiv. 2022:2022.04.08.487609.
15. Krogh A, Larsson B, von Heijne G, Sonnhammer ELL. Predicting transmembrane protein topology with a hidden markov model: application to complete genomes. J Mol Biol. 2001;305(3):567-80.
16. Omasits U, Ahrens CH, Müller S, Wollscheid B. Protter: interactive protein feature visualization and integration with experimental proteomic data. Bioinformatics. 2014;30(6):884-6.
17. Sievers F, Wilm A, Dineen D, Gibson TJ, Karplus K, Li W, et al. Fast, scalable generation of high-quality protein multiple sequence alignments using Clustal Omega. Mol Syst Biol. 2011;7:539.

18. Robert X, Gouet P. Deciphering key features in protein structures with the new ENDscript server. Nucleic Acids Res. 2014;42(Web Server issue):W320-4.
19. Jumper J, Evans R, Pritzel A, Green T, Figurnov M, Ronneberger O, et al. Highly accurate protein structure prediction with AlphaFold. Nature. 2021;596(7873):583-9.
20. Varadi M, Bertoni D, Magana P, Paramval U, Pidruchna I, Radhakrishnan M, et al. AlphaFold Protein Structure Database in 2024: providing structure coverage for over 214 million protein sequences. Nucleic Acids Res. 2024;52(D1):D368-D75.
21. Waterhouse A, Bertoni M, Bienert S, Studer G, Tauriello G, Gumienny R, et al. SWISS-MODEL: homology modelling of protein structures and complexes. Nucleic Acids Res. 2018;46(W1):W296-W303.
